# Source analysis of electrophysiological correlates of beat induction as sensory-guided action

**DOI:** 10.3389/fpsyg.2015.01178

**Published:** 2015-08-14

**Authors:** Neil P. M. Todd, Christopher S. Lee

**Affiliations:** ^1^Faculty of Life Science, University of ManchesterManchester, UK; ^2^Department of Psychology, Goldsmiths CollegeLondon, UK

**Keywords:** sensory-motor integration, rhythm perception, beat induction, vestibular system, source analysis

## Abstract

In this paper we present a reanalysis of electrophysiological data originally collected to test a sensory-motor theory of beat induction (Todd et al., 2002; Todd and Seiss, 2004; Todd and Lee, 2015). The reanalysis is conducted in the light of more recent findings and in particular the demonstration that auditory evoked potentials contain a vestibular dependency. At the core of the analysis is a model which predicts brain dipole source current activity over time in temporal and frontal lobe areas during passive listening to a rhythm, or active synchronization, where it dissociates the frontal activity into distinct sources which can be identified as respectively pre-motor and motor in origin. The model successfully captures the main features of the rhythm in showing that the metrical structure is manifest in an increase in source current activity during strong compared to weak beats. In addition the outcomes of modeling suggest that: (1) activity in both temporal and frontal areas contribute to the metrical percept and that this activity is distributed over time; (2) transient, time-locked activity associated with anticipated beats is increased when a temporal expectation is confirmed following a previous violation, such as a syncopation; (3) two distinct processes are involved in auditory cortex, corresponding to tangential and radial (possibly vestibular dependent) current sources. We discuss the implications of these outcomes for the insights they give into the origin of metrical structure and the power of syncopation to induce movement and create a sense of groove.

## Introduction

### Background and Aims

**FIGURE 1:**
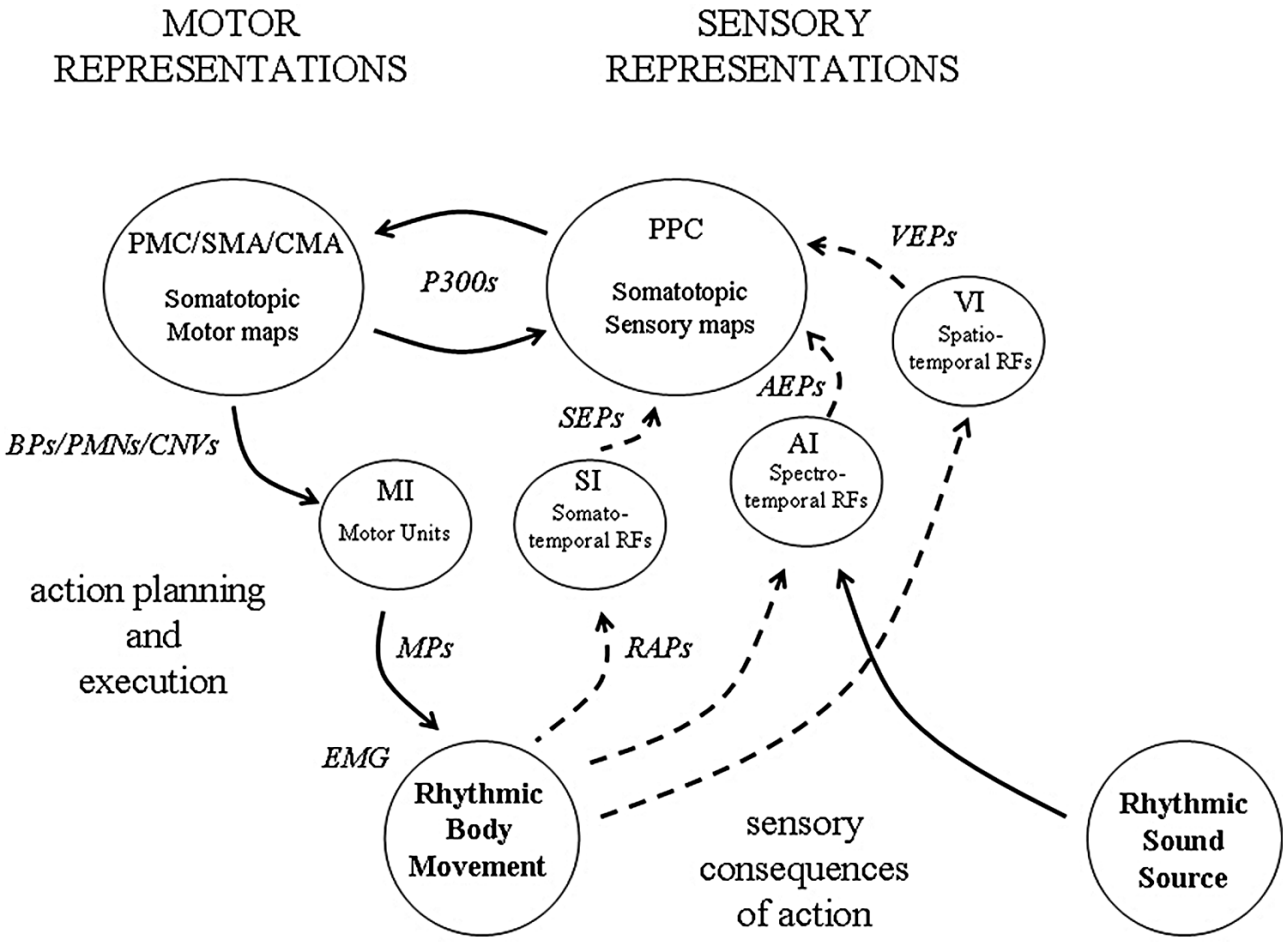
**An illustration of how sensory and motor representations may interact during beat induction.** Neuronal observables are indicated in italic. At the level of the sensory cortex (i.e., AI, VI, SI), receptive fields (RFs) tuned to features of the sensory (i.e., auditory, visual, somatosensory) input represent temporal regularity in the form of a kind of wavelet transform (a form of constant-Q modulation spectrogram). At a macroscopic level, the collective response of populations of such RFs underlies observed evoked potentials (EPs), (i.e., *AEPs, VEPs and SEPs*). At the lowest level in motor cortex (MI) somatotopic representations of the body are organized around motor units, the activity of which can be observed in motor potentials (*MPs*) and *EMG*. Both posterior parietal (PPC) and frontal cortex also contain multiple somatotopic representations of the body which are involved in the planning and execution of movement. These are the loci of P300 potentials, Bereitschaftspotentials (*BPs*), premovement negativities (*PMNs*) and contingent negative variations (*CNVs*). During active synchronization to a rhythmic sound source, external feedback from the sensory consequences of movement becomes available, the origin of the reafference potentials (*RAPs*).

The background to this paper is a theoretical approach which has been referred to as a sensory-motor theory of rhythm perception. Originally conceived some 20 years ago as a result of signal processing experiments on rhythm analysis (Todd and Lee, 1994), the theory developed into an account of how rhythm is processed in the brain (Todd et al., 2002). The essential idea was that the perception and experience of musical rhythm is mediated by two distinct representations – a sensory representation of the auditory input in the form of a kind of wavelet transform, hypothetically represented by receptive fields (RFs) in auditory cortex acting like linear filters, and a sensory-motor representation of the body in frontal and parietal cortex. Reciprocal interactions of these two representations could allow a complex interaction, either by external feedback, i.e., by externally hearing and feeling the consequences of action, or by internal cortico-cortical connectivity (**Figure 1**).

An important development of the theory was a proposal which emerged from an electrophysiological experiment to test the theory (Todd and Seiss, 2004). This was that the results of this experiment could be interpreted as providing evidence for the operation of two distinct sensory-motor circuits: (1) an automatic, internally driven circuit involving pre-motor areas and (2) an attention dependent, externally driven circuit involving posterior parietal areas. The suggestion was made that it was likely that both circuits are co-activated during the presentation of a rhythmic stimulus, but the relative role of the two will depend on the predictability of the rhythm. Subsequent imaging studies essentially confirmed the proposal that rhythmic sequences activate motor areas of the brain (Grahn and Brett, 2007; Schubotz, 2007; Zatorre et al., 2007; Chen et al., 2008), but also that there are two distinct networks (e.g., Lewis and Miall, 2003; Teki et al., 2011; Grahn and Rowe, 2013).

Another important development was the demonstration, consistent with prior predictions (Truslit, 1938; Todd, 1992), that the vestibular system was primal to rhythm perception (Phillips-Silver and Trainor, 2008; Trainor et al., 2009). In a series of experiments initially conducted with infants, evidence was found that bouncing along with auditory rhythms influenced the perception of the rhythmic structure. Similar experiments were conducted with adults, where it was shown that only active and not passive movements had a significant influence on perception. More critically it was demonstrated that head movement and therefore activation of the vestibular apparatus was necessary to observe the effect, and further that vestibular influence could be achieved directly by using galvanic vestibular stimulation (Trainor et al., 2009). In parallel with these behavioral studies, vestibular electrophysiological experiments showed that vestibular receptors contribute to auditory evoked potentials (AEPs; Todd et al., 2003, 2008a, 2014a,b).

In the light of these developments the sensory-motor theory was recast in the form of a “new synthesis” which sought to integrate audio-motor and vestibular perspectives (Todd and Lee, 2015). This new synthesis has a number of implications. One important implication is that there should be an interaction between auditory and vestibular inputs in both the sensory and motor representations during rhythm perception. In this paper we pursue this implication by conducting a reanalysis of the data reported by Todd and Seiss (2004) by means of a vestibular consistent, current source model. Prior to doing so, as a way of preparing the ground for the source analysis, we conduct a review of evoked potential studies which are relevant to the theory of beat induction, and recent vestibular electrophysiological research which inform the interpretation of this data. We also briefly review prior source analysis models and consider how such observed potentials and inferred currents relate to the sensory-motor theoretic constructs described above.

### ERP Studies Related to Beat Induction and Sensory-Motor Synchronization

The use of evoked potentials from averaged stimulus or movement locked EEG is a powerful tool for investigating brain activity associated with sensory and motor behavior, including sensory-motor synchronization and beat induction. We consider studies relevant to the sensory-motor theory and beat induction separately under the categories of AEPs, movement/stimulus preceding potentials and reafference potentials. All three species are integrated during sensory-motor synchronization.

#### Auditory Evoked Potentials (AEPs)

In the case of the auditory system and acoustical stimuli the averaged EEG responses are known as AEPs (Picton, 2011). AEPs come in three species depending on their latency, each of which consist of a series of positive (P) and negative (N) waves: the auditory brain-stem response (ABR), between 1 and 15 ms, indexing activity in the early stages of the pathway; the mid-latency response (MLR), between 12 and 50 ms, consisting of an Na, Pa, Nb, indexing first entry to the cortex; and the long latency response (LLR), from about 50 to 300 ms, consisting of a P1, N1, P2, and also N2 and P3 or P300, depending on the experimental conditions, which are sometimes known as the “cognitive” waves because they are dependent on cognitive factors such as attention. Additional cognitive, more derived potentials with latency similarly to the N1 have been documented, including a mismatch negativity (or MMN) and the emission related N150.

Of the stimulus evoked potentials the cognitive waves, and in particular the P300 and MMN, have proven to be the most revealing in the analysis of rhythm perception and beat induction (Snyder and Large, 2004). The P300 has long been established as a marker of brain responses to violations of expected events, although its interpretation is still controversial (e.g., see Verleger et al., 2005; Verleger, 2010; O’Connell et al., 2012). Its application in a rhythmic context has been demonstrated by a number of researchers, including for subjective accents and omitted events (Brochard et al., 2003; Jongsma et al., 2004, 2005). Similarly to the P300, the MMN has long been established as an electrophysiological marker of violations of prior patterns but is also controversial in its interpretation (Näätänen et al., 2007; Garrido et al., 2009). It is nevertheless considered to be generated in auditory cortex and to represent early sensory processing. In a rhythmic context, several studies have shown sensitivity of the MMN to metrical structure (Vuust et al., 2005; Pablos Martin et al., 2007). Sensitivity to metrical structure can also be demonstrated using MMNs from omission evoked potentials (OEPs; Ladinig et al., 2009), an effect also claimed for neonates (Winkler et al., 2009). It has also been suggested that MMN sensitivity to metrical structure demonstrates that beat induction is automatic and pre-attentive, although this is a matter of some controversy (Bendixen et al., 2009; Geiser et al., 2009; Schwartze et al., 2011; Bouwer et al., 2014; Hove et al., 2014). Some authors have made a finer distinction in the wave components than the above MMN studies, especially between a supposedly pre-attentive N2a and an attention dependent N2b (Schwartze et al., 2011).

#### Movement/Stimulus Preceding Potentials

The classic movement preceding potential is the readiness potential or Bereitschafts potential (or BP; Kornhuber and Deecke, 1990; Shibasaki and Hallett, 2006). Attempts have been made to distinguish various components of it, including an early BP1, from before 500 ms pre-movement, and a late BP2, from after 500 ms pre-movement onset. It has been well established that this potential has its origin in the frontal areas of the brain and in particular in the supplementary motor area (SMA) and cingulate motor areas (CMA), as well as in the motor cortex (MI) in its latter stages, i.e., BP2. There are some differences depending on whether the movement is self-paced or cued (Jankelowitz and Colebatch, 2002).

The contingent negative variation (CNV) has been proposed as an index of brain processes underlying time estimation and is generally observed over frontal regions during the temporal gap between two events, usually a warning and imperative stimulus (Macar and Vidal, 2004; Praamstra et al., 2006). Numerous CNV studies have provided evidence that an integrative process occurs during tempo or time interval encoding which may reflect a memory trace for the time interval (Macar and Vidal, 2003; Pfeuty et al., 2003; Tarantino et al., 2010). A simple interpretation is that the magnitude of the CNV may reflect the size of the temporal interval, although this is controversial because there may be many processes taking place during the comparison (Wiener et al., 2012). It is generally believed that the brain mechanism underlying the CNV operate within the SMA, pre-SMA, or CMA areas (Macar et al., 1999; Mita et al., 2009; Casini and Vidal, 2011), similar to that of the BP, although other areas including parietal cortex are also involved (Wiener et al., 2012).

Although there is now a considerable body of literature on the role of the CNV and other slow potentials in time interval estimation, the application of the method specifically to beat induction in a metrical context has been limited (Zanto et al., 2006). An alternative electrophysiological index which might provide an insight into brain activity prior to a stimulus has been suggested in the form of induced gamma (20–60 Hz) band activity (Pantev, 1995; Bertrand and Tallon-Baudry, 2000). Snyder and Large (2005) found evidence of anticipation in gamma band activity (GBA) using a simple binary rhythm with random omissions on loud or soft tones such that the timing of the GBA on omitted beats was close to the onset of the beat. Zanto et al. (2006) suggested that the generators of GBA might be similar to those of anticipatory slow potentials in frontal cortex, i.e., the BP and CNV, and were thus distinct from the beat following potentials, i.e., MMN and P300.

Iversen et al. (2009) further investigated both gamma and beta (10–30 Hz) MEG activity in passive and imagined beat conditions for simple rhythms formed from two tones with a gap, similar to that used in the Todd and Seiss (2004) studies. They reported evidence that the beta band in particular, the lower end of the Snyder and Large (2005) gamma range (20–30 Hz), was active in subjectively imagined beats. In their interpretation they went on to describe a sensory-motor theory, consistent with that proposed by Todd and Seiss (2004), i.e., that beat induction is mediated by the interaction of motor and sensory components, and not by an attentional system. In support of their proposal they were able to cite imaging data which was by then available, implicating motor areas in rhythm perception (see Todd and Lee, 2015 for review), and additional data which indicated a beta oscillation network in sensory-motor synchronization (Pollok et al., 2005). The beta oscillations, it was suggested, allowed the synchronized coupling of the various sensory and motor areas.

Fujioka et al. (2009) examined both beta (15–30 Hz) and gamma oscillations (>30 Hz) in the auditory cortex to rhythms composed of loud and soft tones. They reported that beta activity synchronized with an isochronous sequence, decreasing after tones, but increased “excessively” after an omitted tone. In contrast the gamma activity peaked 80 ms after a present tone, but 110 ms after an omitted tone. They suggested that the two oscillation bands subserved two distinct functions. Whereas the gamma band in their view reflected an endogenous process of anticipatory entrainment, the beta band reflected an exogenous audio-motor coupling process (Fujioka et al., 2010). More recently Fujioka et al. (2012) applied a beta band analysis to isochronous sequences of different rates suggesting the beta “rebound” between stimuli related to an interval timing process. They also carried out a coherence analysis which implied various motor areas had become coupled to the auditory activity.

#### Reafference Potentials

The third set of electrophysiological components relevant to the sensory-motor approach to beat induction is the reafference potentials (RAPs), of somatosensory origin, which are a consequence of movement. Such potentials can be observed following the BP, usually in the form of post-motion potentials of both negative and positive polarity (Kornhuber and Deecke, 1990; Shibasaki and Hallett, 2006). The origins of such potentials are likely to arise from both proprioceptive receptors, such as muscle spindles, and tactile receptors within the skin, and their projections to somatosensory cortex. Somatosensory evoked potentials (or SEPs), produced by passive vibration or movement have been well-studied and characterized (Todd et al., 2014c). Typically SEPs are characterized by a main positive peak recorded contralaterally at about 50 ms following finger movement (Hämäläinen et al., 1990). This peak is also analogous to an N60 peak recorded following electrical stimulation of the median nerve.

#### Sensory-Motor Synchronization

All three of the above species of potentials come together within the realm of sensory-motor synchronization (Müller et al., 2000). A study of error correction in sensorimotor synchronization by Praamstra et al. (2003) using EEG demonstrated examples of stimulus and movement related potentials. Essentially there are two morphologies, depending on whether the averaging is done relative to the stimulus during passive listening or the movement during synchronization: (a) stimulus related potentials, consisting of the P1, N1, P2, and N2 waves, as above, and (b) movement related potentials, consisting of a PMN, equivalent to the BP, a re-afferance negativity (RAN) and a post-motion positivity (PMP; Jankelowitz and Colebatch, 2002). Third hybrid morphology is obtained when locking the averaging to the stimulus during active synchronization.

### Vestibular Receptors Contribute to Auditory Evoked Potentials

#### Vestibular Evoked Myogenic Potentials (VEMPs)

As reviewed in Todd and Lee (2015), there have been in the last two decades dramatic changes in vestibular clinical neurophysiology with the discovery of a sound evoked potential referred to as a vestibular evoked myogenic potential (VEMP; Colebatch et al., 1994). It was established that the VEMP was a manifestation of the vestibular-collic reflex mediated by acoustic sensitivity of the otolith organs and the vestibular spinal tract. Its vestibular dependency was established by the fact that it was absent in avestibular patients but could be measured in the deaf with normal vestibular function. As well as being a clinical tool, the VEMP could also be used as a scientific tool to investigate the acoustic sensitivity of the otolith organs to both air- and bone-conducted sound. In a series of papers, Todd and Cody (2000), Todd et al. (2000), Todd (2001) at Manchester showed that VEMPs from air-conducted sound were tuned with a best frequency of about 500 Hz, that they could be activated by sounds found in the environment, such as at musical concerts, when stimuli were above about 80 dB SPL and that there was a hedonic response to acoustic vestibular sensations.

Subsequently another sound-evoked myogenic response was discovered, but this time a manifestation of the vestibular ocular reflex (VOR; Rosengren et al., 2005, 2010). From this emerged the ocular VEMP or OVEMP (Todd et al., 2004, 2007; Todd, 2010, 2013). Apart from the clinical work, the OVEMPs also provided a useful tool to investigate vestibular sensitivity. In a series of tuning studies Todd et al. (2008b, 2009) found that in addition to the 500 Hz sensitivity to AC sound, there appeared to be another lower-frequency sensitivity, especially to vibration at about 100 Hz. They suggested that this may reflect the different biomechanical properties of the saccule and utricle. Later studies have shown that both sound and vibration vestibular tuning curves show two peaks, at about 100 and 500 Hz (Zhang et al., 2011, 2012).

More recently several studies have been done which suggest that the VEMP threshold to AC sound may be lower than previously suggested (Dennis et al., 2014). This is in part because the VEMP magnitude obeys a power law and there is always a signal to noise ratio issue when finding thresholds. Further the VEMP threshold will always be higher than the acoustic receptor threshold, due to attenuation in the reflex pathway that mediates the VEMP. It is likely therefore, that the receptor rate threshold may be as low as 70 dB SL or less, and the phase-locking threshold, which is about 10 dB below the rate threshold, as low as 60 dB SL (McCue and Guinan, 1994; Todd et al., 2014b).

#### Vestibular Evoked Potentials of Neurogenic Origin (VsEPs)

In parallel with the above work on VEMPs, efforts were made to look for vestibular evoked potentials of central neurogenic origin, referred to as vestibular evoked potentials (or VsEPs). Following an evoked potentials study using galvanic stimulation of the exposed vestibular nerve with Ménière’s disease patients (de Waele et al., 2001), new short-latency VsEPs were found using bone-conducted sound (Todd et al., 2003; Rosengren and Colebatch, 2006), which were related to the OVEMPs. Subsequently Todd et al. (2008a) described (using higher resolution analyses) short latency VsEPs, from 10 to 30 ms. Most recently this work was extended to longer latencies where it has been shown that vestibular receptors contribute to long latency AEPs (Todd et al., 2014a,b).

### Source Analysis of Beat Induction Related Potentials

Of particular importance to the interpretation of evoked potentials are techniques which allow an estimation of the neural activity underlying them, otherwise known as the “inverse problem.” The basic principle is that the measured scalp voltages should be explained by electrical current sources within the brain. There are a large number of solutions to this problem, which may be broadly classified as parametric and non-parametric (Grech et al., 2008). The essential difference between the two approaches is that whereas the parametric approach starts with a fixed number of electric dipoles, and searches for the best fitting location and orientation of dipoles, the non-parametric approach sets out to determine an underlying continuous current density within the brain volume without assuming any fixed number of sources. An exemplar of the parametric approach is brain electrical source analysis (or BESA; Scherg and Berg, 1991), and for the non-parametric approach, low resolution electromagnetic tomography (or LORETA) (Pascual-Marqui et al., 1994). Non-parametric approaches are preferred by many practitioners as they require fewer assumptions and BESA requires some user expertise, e.g., as the outcomes are dependent on the initial sources. There are though some important limitations on LORETA, one being that the estimated current density solution is constrained to cortical gray matter. From a vestibular perspective this limitation is critical as sub-cortical and extra-cephalic sources, e.g., from the musculature around the head and eyes, are likely involved. For this reason our method of choice is BESA.

Brain electrical source analysis has been applied with some success to each of the species of potential described above. For the late AEPs N1/P2 from the earliest it was proposed that they were generated by bilateral tangential and radial components in primary and secondary auditory cortex (Scherg and Von Cramon, 1986; Näätänen and Picton, 1987; Scherg et al., 1989). Radial and tangential components may be combined into bilateral regional sources. The existence of these components has been confirmed in many subsequent studies and is the generally accepted standard model of the N1/P2 (e.g., Hegerl et al., 1994; Shahin et al., 2003, 2007). However, additional generators in the frontal and cingulate cortex have also been implicated (Alcaini et al., 1994; Giard et al., 1994). These additional generators have been associated variously with brain catecholamine activity, affective dysfunction or auditory cognitive function more generally (Gallinat et al., 2000, 2002; Juckel et al., 2003; Bender et al., 2006).

Brain electrical source analysis applied to short-latency VsEPs produced from sound indicates that these are dominated by the VOR, and the central brain structures which are involved in VOR control, including the brain-stem/cerebellum (Todd et al., 2008a). Most recently BESA applied over a longer time epoch confirmed that the short-latency responses are dominated by ocular/cerebellar effects but also indicated a large cingulate cortex source and a contribution from the STG (Todd et al., 2014a,b). Of particular relevance here was the reported suggestion that there was a vestibular contribution to the radial component of the N1. The presence of vestibular projections to the temporal lobe confirmed that the auditory and vestibular pathways are much more entwined than hitherto suspected. A vestibular dependence of these components is consistent with their prominence in studies on the loudness dependence of AEPs which make use of high intensities up to 100 dB SPL (Gallinat et al., 2000), likely well above the vestibular threshold.

Source analysis of the BP confirms the involvement of SMA and bilateral MI in its generation (Praamstra et al., 1996; Erdler et al., 2000). In addition such models also implicate a somatosensory source corresponding to the reafference following the movement (Praamstra et al., 1996). Source analysis of SEPs using a four dipole model indicates that the SEP N60 from median nerve stimulation has a large contribution from a radial source in the postcentral gyrus contralateral to the stimulated hand (Srisa-an et al., 1996). This peak has also been attributed to both radial and tangential components originating from two perirolandic dipoles (Barba et al., 2002). However, Barba et al. (2002) also reported frontal sources, suggesting both somatosensory and motor generators of the SEPs. Thus both the BP and SEP contain both sensory and motor components.

### From Sensory-Motor Theoretic Constructs to Observed Sources

As noted above the sensory-motor theory of beat induction proposes that our experience of a rhythm results from the reciprocal interaction of a sensory representation of the auditory input in the form of a kind of wavelet transform, mediated by a population of band-pass filters likely represented in auditory cortex, and a sensory-motor representation of the body likely located in frontal/parietal areas. The wavelet-like transform can be thought of as a kind of running log-scale modulation spectrogram which allows tempo and rhythm pattern recognition. A particular rhythmic pattern will remain invariant despite changes of tempo, only shifted up or down in scale space. An important question is how these concepts relate directly to the observed potentials and estimated source currents described in the preceding section. **Figure 1** represents how we believe these might be linked.

Considering first sensory representations, as we have described in more detail elsewhere (Todd, 1999; Todd et al., 1999), the neural basis of auditory rhythm perception can be seen to be analogous to that of the visual motion detection system within the visual cortex (Clifford and Ibbotson, 2003). In the visual case the spatio-temporal power spectrum of the retinal image as projected to cortex is sampled by a population of RFs which may be described mathematically in the form of Gabor-like filters tuned to a range of spatial and temporal frequencies (Heeger, 1987). Each of the RFs is instantiated within a local collection of primary visual cortex (VI) cortical columns receiving inputs from the thalamus and reciprocal cortico-cortical connections from other neighboring or remote sites. A secondary population of RFs within secondary visual areas are involved in processing the outputs of the primary RFs. The properties of individual RFs can be investigated by means of single unit recordings in animal preparations.

In human studies the visual motion system can be investigated by means of surface EEG/MEG and the production of motion visual evoked potentials (VEPs) from motion stimuli, such as sinusoidal gratings in which both a spatial frequency (cycles per degree) and temporal frequency (cycles per second) is specified (Heinrich, 2007). Motion VEPs like AEPs consist of a sequence of positive and negative waves in the range 50–400 ms, but are dominated by a negativity, an N2 at about 200 ms (Heinrich, 2007). Although the details of the link between the activity of the many 1000s of individual RFs in response to a stimulus and a VEP is not fully described it is generally agreed that the VEP represents the collective behavior of the entire distributed population of RFs. A source analysis of motion VEPs reveals a sequential activation of currents V1 and secondary areas consistent with our understanding of motion processing at the level of RFs (Pitzalis et al., 2012). Despite the absence of a well-described RF/VEP linking mechanism, the VEP shows properties which reflect those of the RF population in terms of sensitivity to spatial and temporal frequency.

As with visual motion, so too with auditory rhythm. RFs in auditory cortex responsive to amplitude and frequency modulation have been described since the earliest days (Whitfield and Evans, 1965). The existence of complex spectro-temporal RFs and their modeling is now well-advanced (Schreiner and Mendelson, 1990; Shamma and Versnel, 1995; Kowalski et al., 1996). Thus in the same way that VEPs can be considered representative of a RF population response, AEPs also may be considered the collective and summed response of a population of auditory cortical RFs tuned to spectral and temporal frequencies, despite the lack of precise knowledge of the linking mechanism between activity at the level of the single RF and the output and interaction of the entire population. Under appropriate experimental conditions AEPs will exhibit properties reflecting the response of the underlying RF population to stimulus rate. Our theory predicts further that should stimulus intensity exceed the vestibular threshold then the AEPs will additionally show a rate sensitivity property which is consistent with that of the vestibular system. Thus auditory rhythm perception should show a vestibular interaction which parallels the visual-vestibular interaction which takes place in the visual motion system (Smith et al., 2012).

There is another sense in which the visual motion and auditory rhythm mechanisms share a parallelism, which is in the distribution of the RF temporal components. It is well-established that the visual motion system is partitioned into slow and fast motion sub-systems (Anderson and Burr, 1985). The slow motion system is characterized by a population of RFs which have temporal low-pass filtering characteristics, while the fast system is characterized by a population of RFs possessing a band-pass characteristic. Collectively the slow and fast motion systems reflect these low-pass and band-pass properties. Elsewhere we have proposed that within the auditory system rhythmic grouping structure perception is mediated by a population of RFs which have a low-pass filter characteristic (Todd, 1994; Todd et al., 1999). This population is distinct from the population of auditory cortical RFs which have band-pass filter characteristic and which mediate metrical structure perception.

Of particular interest is the fact that the temporal frequency distribution of the visual motion and auditory rhythm RFs overlap considerably in the range ∼0.5–16 Hz (Anderson and Burr, 1985; Hess and Snowden, 1992). This should not be surprising when we consider that the underlying object movements which generate common audio-visual phenomena, such as lip movement during speech, are the same (McGurk and MacDonald, 1976). It is natural, therefore, that RFs in auditory and visual cortex should be similarly tuned as they represent the temporal power spectrum of the same natural change of objects in the environment, either through the object’s modulation of sound or reflected light (Fujisaki and Nishida, 2005). The commonality of auditory and visual RF temporal properties also provides the temporal coherence basis for them to bind together via cortico-cortical connections between RFs, thus forming a single neuronal assembly underlying an audio-visual percept (Engel et al., 2012).

The above mechanism of audio-visual binding through temporal coherence naturally extends to sensory-motor representations of the body in the brain (Engel et al., 2012). Somatotopic organization of the somatosensory (SI) and motor (MI) cortices are, of course, well established (Flanders, 2005). However, the somatotopic principle extends to secondary sensory and motor areas in premotor or CMA/SMA and posterior parietal (PPC) areas (Muakkassa and Strick, 1979; Eickhoff et al., 2008). There are, therefore, multiple somatotopic representations of the body in sensory and motor cortices. In the same way that visual and auditory areas can be broken down to a larger number of smaller individual RFs, each of the somatotopic maps can also be said to be composed of a number of RFs which represent smaller regions of the body. In the case of the somatosensory system RFs are characterized by small touch zones of the body surface. In the case of the motor system a RF can be thought of as corresponding to an individual motor unit. Again by analogy to the link between the collective response of a population of visual or auditory RFs to the summated response represented by VEPs or AEPs, the same is true of the relationship between sensory or motor RFs and SEPs or BPs, although they are likely to be more localized to a specific part of the body, e.g., from the vibrotactile stimulation or movement of a single digit. To complete the present account of the link between sensory-motor theoretic constructs and observed potentials and inferred source currents, however, it should be noted that, in addition to their modality specific properties, such sensory and motor RFs must also be attuned to the temporal regularities of their input/outputs. Thus a cortico-cortical binding from temporal coherence is also possible between auditory/visual and somatosensory or motor RFs (Engel et al., 2012).

To summarize this position (see **Figure 1**), auditory, visual, somatosensory and motor cortex are highly organized into populations of RFs which represent smaller features within cochleotopic, retinotopic, and somatotopic maps. Evoked potentials from averaged EEG/MEG produced by modality specific stimulation correspond to the collective output of a population of RFs activated by the particular stimulus. Although RFs each represent distinct modalities they must also represent temporal regularities which are not modality specific but which are common across modalities, such as those that occur during synchronization. Such temporal regularities give rise to temporal coherence and allow the formation of multi-modal sensory-motor neuronal assemblies. The observed potentials and inferred currents represent the population response of those individual RFs which form the assembly.

## Source Analyses of Electrophysiological Correlates of Beat Induction

Having reviewed in the previous section the neurophysiological background to the sensory-motor theory of rhythm, and described how the concept of RFs relates to observed scalp potentials and underlying currents, we return to the electrophysiological data collected by Todd and Seiss (2004). In this section we provide further motivation for the revisitation, give a representation of the methods, and then conduct a source analysis of the data, followed by a discussion of the source analysis results. A more general discussion of the implications of these data is reserved for the general discussion.

### Motivation

In the preceding section it was noted that AEPs have their genesis in both temporal and frontal generators. Furthermore, such generators are both intensity and vestibular dependent. Similarly it was also noted that both movement related potentials and reafferance potentials may have both motor and somatosensory generators. Thus during auditorily cued synchronization we should expect sensory and motor areas to be reciprocally activated (**Figure 1**), and increasingly so with higher intensity auditory stimuli which progressively recruit vestibular receptors. For a specifically rhythm perception task, where there may be no overt movement, such reciprocity is likely to be critical to our understanding of beat induction from the perspective of the sensory-motor theory. Of particular interest is the possibility that during a beat induction task acoustic activation of vestibular receptors is central because the auditory cortical and cingulate sources described for vestibular contributions to AEPs are in close proximity to the areas associated with beat induction.

As also noted in the introduction (and see Todd and Lee, 2015) there is now considerable evidence that vestibular inputs do indeed contribute to rhythm perception. It is therefore quite possible that a number of the beat induction studies cited above may have included hitherto unrecognized vestibular components, not least the Todd and Seiss (2004) study which made use of loud click stimuli. This impression is strengthened by the fact that the source analyses given in Todd and Seiss (2004) and in Todd et al. (2014b) are similar. In particular both analyses include a radial temporal lobe component to explain the later part of the N1 wave and a cingulate cortex source. For this reason in this section we reanalyze the 2004 data using source analysis procedures developed for VsEPs. The aim of the reanalysis is to investigate the pattern of activity in current sources in the areas of interest produced during a beat induction task.

**FIGURE 2:**
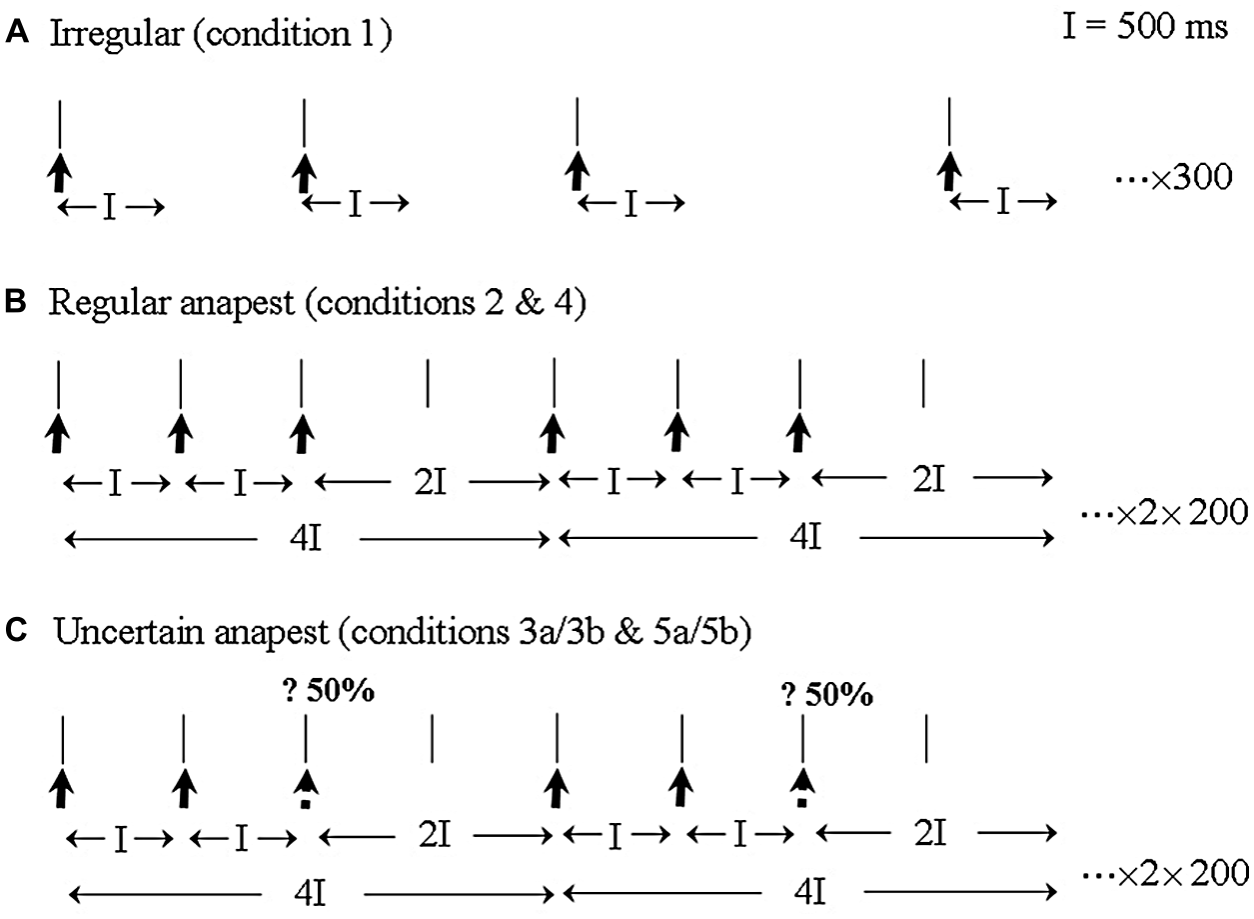
**An illustration of the three main stimulus categories and EEG recording epochs. (A)** *The irregular rhythm* (condition 1) consisted of a sequence of clicks with random inter-click intervals between 1000 and 2000 ms. EEG was sampled continuously with an averaging epoch of 500 ms. **(B)** *The regular anapest* [condition 2 (passive) and 4 (active)] consisted of three clicks and a gap with inter-onset intervals of 500, 500 and 1000 ms, which metrically corresponds to a bar of “Three blind mice.” EEG was epoched for averaging over the whole duration of the anapest rhythm, including for the last long interval which acoustically contains no click but perceptually contains a weak beat. **(C)** *The uncertain anapest* [condition 3 (passive) and 5 (active)] consisted of the same pattern as the regular anapest except that on 50% of presentations the third click was absent. On those presentations with the third click absent the stimulus was syncopated [condition 3a (passive) and 5a (active)] and on those with the third click present the stimulus was unsyncopated [condition 3b (passive) and 5b (active)]. The EEG recording epoch was identical to that of the regular anapest.

The essential idea behind the Todd and Seiss (2004) study was to adapt the experimental procedure described by Praamstra et al. (2003) for synchronization to an isochronous tone sequence for use with a non-isochronous rhythmic pattern. Musically trained subjects were required to listen to an anapest rhythm, consisting of three clicks, with inter-onset intervals of 500 ms, followed by a gap of 1000 ms, under active and passive conditions. The anapest was preceded by a condition in which the stimulus was unpredictably irregular and followed by a syncopation condition which introduced uncertainty as to the presence or absence of the third click (see **Figure 2**). For convenience we reproduce here the methods section from Todd and Seiss (2004).

### Methods

#### Subjects

Ten musically trained subjects were selected from staff and students at the University of Birmingham. Musicians were chosen as subjects because we expected musically untrained subjects to be less proficient in the synchronization task and the data to be more noisy. The subjects played a variety of instruments, but none were percussionists. They were all right handed and had no hearing or cognitive impairments. Prior to any testing, all participants gave written informed consent according to the Declaration of Helsinki. The University of Birmingham Research Ethics Committee approved the study.

#### Stimuli

Stimuli were 1 ms, compressive square pulses, producing a robust click, presented at a loud but not uncomfortable intensity, about 80–90 dB SPL.

#### Apparatus

EEG, EMG, and EOG were recorded continuously with Ag/AgCl electrodes located in scalp, muscle and periocular positions. Finger movements were recorded using a force-plate transducer. EEG locations were given by the 10-5 extension of the 10–20 system (Oostenveld and Praamstra, 2001). EMG was recorded differentially in positions above the flexor and extensor indicis muscles. All signals were amplified with a band pass of 0.16–128 Hz by BioSemi Active-One amplifiers and sampled at 512 Hz. Stimuli were generated using Eprime software and delivered by detachable headphones placed under the cap.

#### Procedure

After preparation, subjects were seated in a comfortable chair and were given instructions. They were told that they would be presented with a number of rhythms in blocks lasting about 4 min. At the start of each block they would be given a warning tone. During the blocks they were to be as relaxed as possible and avoid unnecessary movements with gaze fixated but to maintain attention on the rhythm. Recordings were arranged in five sets consisting each of two blocks.

For the first three sets the three stimulus categories (as in **Figure 2**) were presented in a passive listening mode. A *passive irregular* condition (condition 1), consisted of 300 repetitions of a click in which the inter-onset interval of the clicks varied randomly between 1000 and 2000 ms with a rectangular probability distribution. A *passive regular* condition (condition 2), consisted of 200 cycles of an anapest rhythm made up of three clicks with inter-onset interval of 500 ms followed by a gap of 1000 ms. Passive *uncertain* conditions (condition 3) consisted of 400 cycles of the regular anapest rhythm randomly alternated with a syncopated rhythm consisting of two clicks with inter-onset interval of 500 ms followed by a gap of 1500 ms. There were thus two sub-conditions, i.e., syncopated (condition 3a) and unsyncopated (condition 3b). Both were presented with equal probability (200 cycles each).

In the last two sets the second and third stimulus categories (as in **Figures 2B,C**) were presented in an active listening mode. An *active regular* condition (condition 4), consisted of the same stimulus as condition 2, but where the subjects synchronized the extension of their right index finger with the beat of the regular anapest rhythm. A*ctive uncertain* conditions (condition 5), consisted of the same stimulus as condition 3 but where subjects synchronized to the beat of the uncertain anapest rhythm. Again there were two subconditions, active syncopated (condition 5a) and active unsyncopated (condition 5b), (there being thus seven rhythm conditions in total). The movement was a flick without using any force on the return and without movement of the other fingers. This was to avoid significant activity in the flexor muscle. The movement target was a tap on a force-plate which was adjusted to be about 2 cm above the index finger which at rest hung loosely over the end of the arm rest.

#### EEG/EMG Analysis

Continuous EEG recordings were segmented off-line into epochs of 2000 ms starting 100 ms before the first click of the rhythm. For the irregular rhythm epochs had a length of 600 ms and started 100 ms before the tone. The epochs were shorter in the irregular condition as they only contained a single click. Epochs containing significant artifacts were rejected from the analysis. Using these epochs evoked potentials from averaged EEG were obtained for each of the rhythm conditions for each subject. Subsequently grand means of evoked potentials across subjects were obtained for each rhythm condition. For the uncertain rhythm conditions the averaging was carried out separately for those trials with the click on the third beat present (condition 3b and 5b) and those with it missing (condition 3a and 5a). Finger EMG was rectified, low-pass filtered at 128 Hz and averaged in the same way. Due to excessive artifact and low signal-to-noise ratios, data from two of the subjects were not included in the final analysis.

#### Brain Electrical Source Analysis

Brain electrical source analysis software (version 5.1 MEGIS Software GmbH, Germany) was used for dipole modeling. The standard four-shell elliptical head approximation was employed with the following parameters. The radial thickness of the head, scalp, bone and CSF were 85, 6, 7, and 1 mm respectively with conductivities set to 0.33, 0.33, 0.0042, and 1.0 respectively. The modeling approach adopted here differs to that of the 2004 study, where narrow fitting intervals focused on the individual components were used, in that we take the whole epoch as the fitting interval. In addition we make use of paired symmetrical regional sources for the auditory cortex component, rather than modeling the tangential and radial components separately. We also apply a strict acceptance criterion that a model is only accepted if the regional sources locate proximal to within 5 mm of BA 41 and 42 which constitute the transverse temporal gyrus containing primary auditory cortex. This modeling strategy was found to be effective and robust for a number of data sets (e.g., see Todd et al., 2014b).

### Results of the Source Analysis

In the following subsections we apply the approach described above to give a description of the source analysis results for each of the rhythm conditions after band-pass filtering between 0.2 and 30 Hz (zero phase, 12 dB/octave slope) within the BESA software. However, rather than repeat the fitting procedure independently for each case, we first apply it in detail to the random case (condition 1), where we show a small number of alternative models, and then for one selected model, chosen for plausibility and goodness of fit, we apply it unchanged to all other conditions. There was not any obvious qualitative difference between the active regular case (condition 4) and active uncertain case (condition 5) so we only show below the details of the active regular case. A quantitative statistical analysis of these results is subsequently presented in the next Section “Quantitative Testing of the Results.”

#### Passive Listening to an Irregular Rhythm (Condition 1)

**FIGURE 3:**
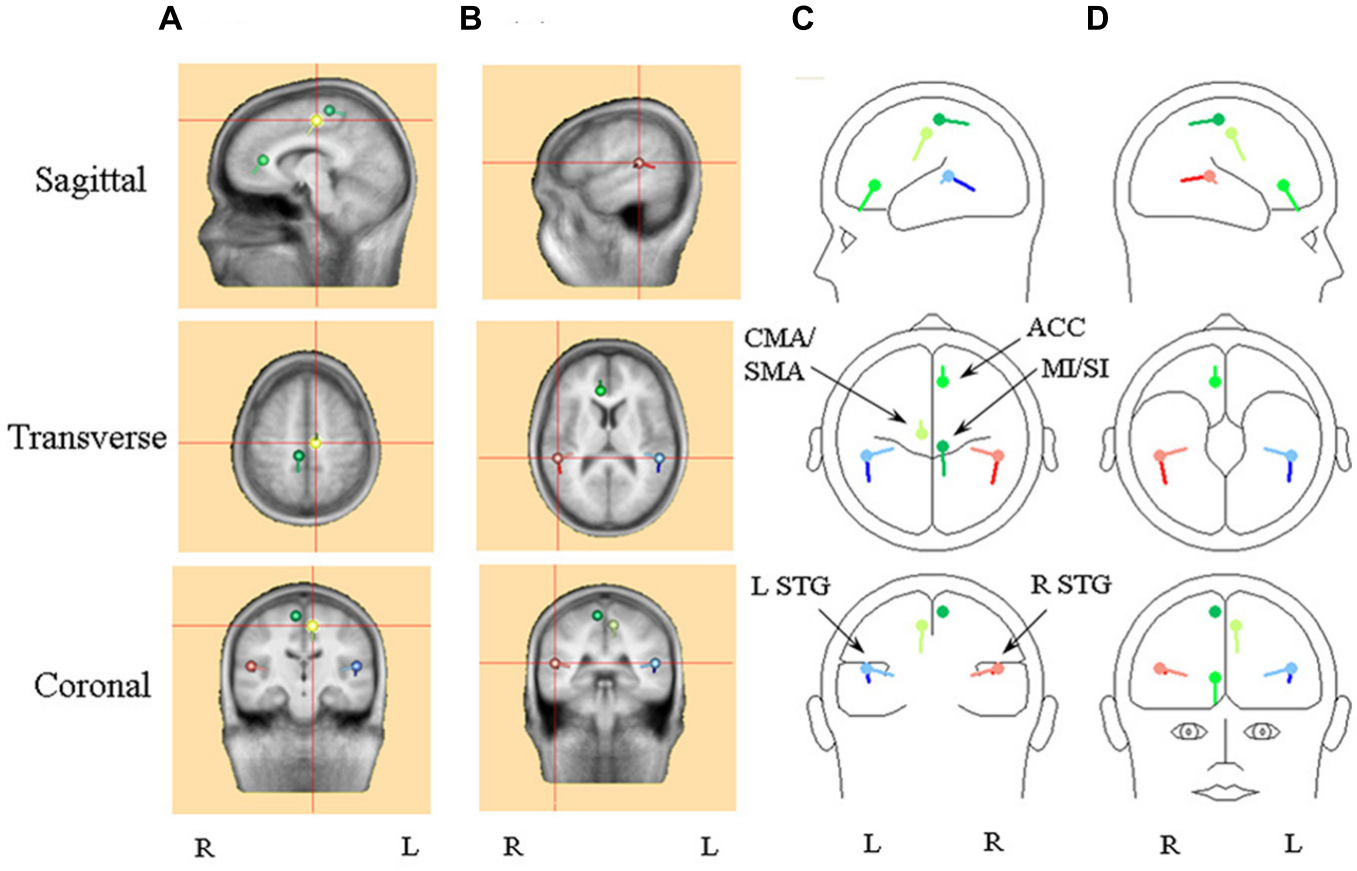
**An illustration of the optimum source model selected for detailed comparison, i.e., the model with the pair of regional sources and three dipole.** Frontal sources are illustrated with a shade of green, with the cingulate/supplementary motor area (CMA/SMA) source distinguished by light green and the primary sensory-motor cortex source (SI/MI) by dark green. Right hemisphere superior temporal (STG) sources are in red, with the radial source distinguished by light red. Left hemisphere STG sources are in blue, with the radial source distinguished by light blue. **(A)** Standard MRI images centered on the CMA/SMA source. **(B)** Standard MRI images centered on the right STG sources. **(C)** A BESA representation of the sources shown from left sagittal, superior and posterior perspectives. **(D)** A BESA representation of the sources shown from right sagittal, inferior and frontal perspectives.

**Table 1** provides the Talairach–Tourneaux coordinates (TTCs) for a number of models which meet the criterion of the regional sources locating to within 5 mm of BA 41/42 when applied to the random condition. In addition to bilateral auditory cortex the solutions all contain at least one midline source which is close to the cingulate cortex (BA 23/24/31), and in models with more than one additional source, they are close to or within the MFG in BA 6. The optimal source model selected for detailed comparison is that with the pair of regional sources and three midline dipoles, illustrated in **Figure 3**. This was chosen for having the lowest residual variance, which was 2.6%. The three midline frontal sources are hereafter labeled as anterior cingulate (or ACC), cingulate motor/supplementary motor area (or CMA/SMA) and sensory-motor cortex (or MI/SI). When the temporal lobe sources (STG) are broken down into their individual components, in addition to prominent tangential components (tSTG), there are also prominent radial components (rSTG). Although employing the more robust fitting method, these solutions are in fact similar to those published in 2004 using the more piecemeal approach. For comparison, the solutions for evoked potentials produced by an explicitly vestibular stimulus (from Todd et al., 2014b) are also shown in **Table 1** which illustrate the similarity of the present solutions of the 2004 data, including the optimal source model, to the Todd et al. (2014b) vestibular solutions. In particular all solutions include both tangential and radial components of the STG sources and a prominent midline source within the cingulate region. One important difference, though, is that the Todd et al. (2014b) solutions include cerebellar and ocular sources which are not present in the optimum source model of the Todd and Seiss (2004) data. The essential reason for this difference is due to the difference in sampling rates employed, 512 Hz versus 8 kHz respectively for the Todd and Seiss (2004) and Todd et al. (2014b) data. Both the ocular and cerebellar responses occur rapidly, within about 20 ms, and are therefore unresolved at the slower sampling rate.

**Table 1:** Talairach–Tourneaux coordinates (TTCs) for source models for random condition. (Filters 0.2–30 Hz, interval 25–425 ms).

Model	Date	*X*	*Y*	*Z*	RV	Region	BA
RS1&2 1DP	2004	±40 -4	-27 -7	20 37	4.0	Ins/STG CG	13/41 24/23
RS1&2 DP1&2 DP3	2004	±51 ±8 9	-37 -25 23	11 56 14	2.7	STG/MTG MFG/PCG ACC/Caudate	41/22/42/21 6/5 24/33
RS1&2 DP1 DP2 DP3	2004	±50 -8 7 8	-33 -18 -29 34	12 49 60 12	2.6	STG/TTG MFG/PCG PCG/MFG ACC	41/42 6/31 6/3/4 24/32
RS3&4 DP1&2 RS1&2	2014b Left	±52 ±4 ±39	-18 4 58	3 40 -39	2.4	STG/Ins CG/MFG EOM	22/41/21/13 24/32
RS3&4 DP1&2 RS1&2	2014b Right	±51 ±4 ±36	-16 -6 60	4 38 -36	2.1	STG/Ins CG EOM	22/41/13/21 24/31

*ACC, anterior cingulate gyrus; BA, brodmann area; CG, cingulate gyrus, DP, dipole; Ins, insula; EOM, extra ocular eye muscles; IFG, inferior frontal gyrus; L, left; MFG, medial frontal gyrus; MTG, medial temporal gyrus; PCG, precentral gyrus; RS, regional source; RV, residual variance; SFG, superior frontal gyrus; STG, superior temporal gyrus; TTC, Talairach–Tournoux coordinates; TTG, transverse temporal gyrus.*

**FIGURE 4:**
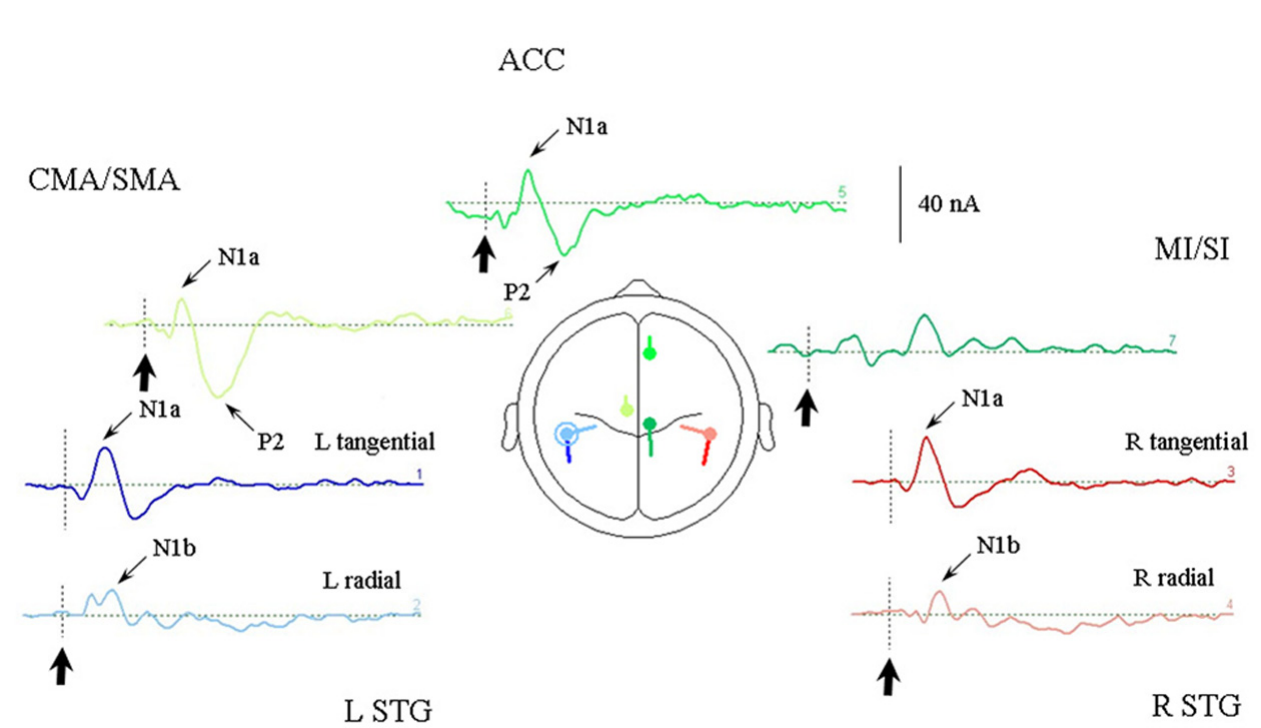
**Response of the optimum source model sources to the irregular rhythm (condition 1).** Each of the seven sources are illustrated in the same colors as given in **Figure 3** and arranged in approximate location relative to a superior transverse perspective. The stimulus onset is indicated by bold arrows. Sources contributing to the AEP N1a, N1b, and P2 are indicated. Note that the largest STG contribution to the N1a is from the tangential sources, and to the N1b by the radial sources.

One other feature which the optimum source model of the Todd and Seiss (2004) data has in common with the Todd et al. (2014b) solutions is that each source component may contribute to each of the measured potentials, which is not the case in the piecemeal approach of the older solutions suggested in Todd and Seiss (2004). This is clear from **Figure 4**, which illustrates the model response to the irregular stimulus, and **Table 2** where the relative contributions of the sources from the optimum source model are given at the sampling points. The sources are labeled according to their location and or orientation within the temporal or frontal lobes. The N1a receives the largest contribution from the tangential STG (or tSTG) sources of about 20 nA, but also from ACC and CMA/SMA sources of about 10 nA. Similarly the N1b receives a large contribution from the radial STG (or rSTG) sources, but a further contribution of a similar magnitude from the CMA/SMA source. The P2 shows a similar pattern of source contributions as for the N1a, except that here the ACC and CMA/SMA sources at 30 nA are larger than the tSTG sources of about 15 nA. The largest contributor to the N2a and N2b comes from the MI/SI source, but with the N400 receiving a more even contribution from all sources.

**Table 2:** Source current strengths (nA) for the optimum source model.

Source	N1a (95 ms)	N1b (130 ms)	P2 (180 ms)	N2a (260 ms)	N2b (300 ms)	N400 (390 ms)
L STG (tan.)	-18	-6	+16	+4	+1	-3
R STG (tan.)	-22	-6	+13	+4	-1	-4
L STG (rad.)	-5	-12	+5	+6	+1	+3
R STG (rad.)	+4	-12	+5	+5	+4	+8
ACC	-11	-4	+29	+5	+6	+1
CMA/SMA	-12	+12	+34	+7	-5	-3
MI/SI	-6	-3	-1	-10	-14	-5

*ACC, anterior cingulate gyrus; CMA, cingulate motor area; MI, primary motor area; SI, primary somatosensory area; SMA, supplementary motor area; STG, superior temporal gyrus; RS, regional source.*

#### Passive Listening to a Regular Anapest (Condition 2)

**FIGURE 5:**
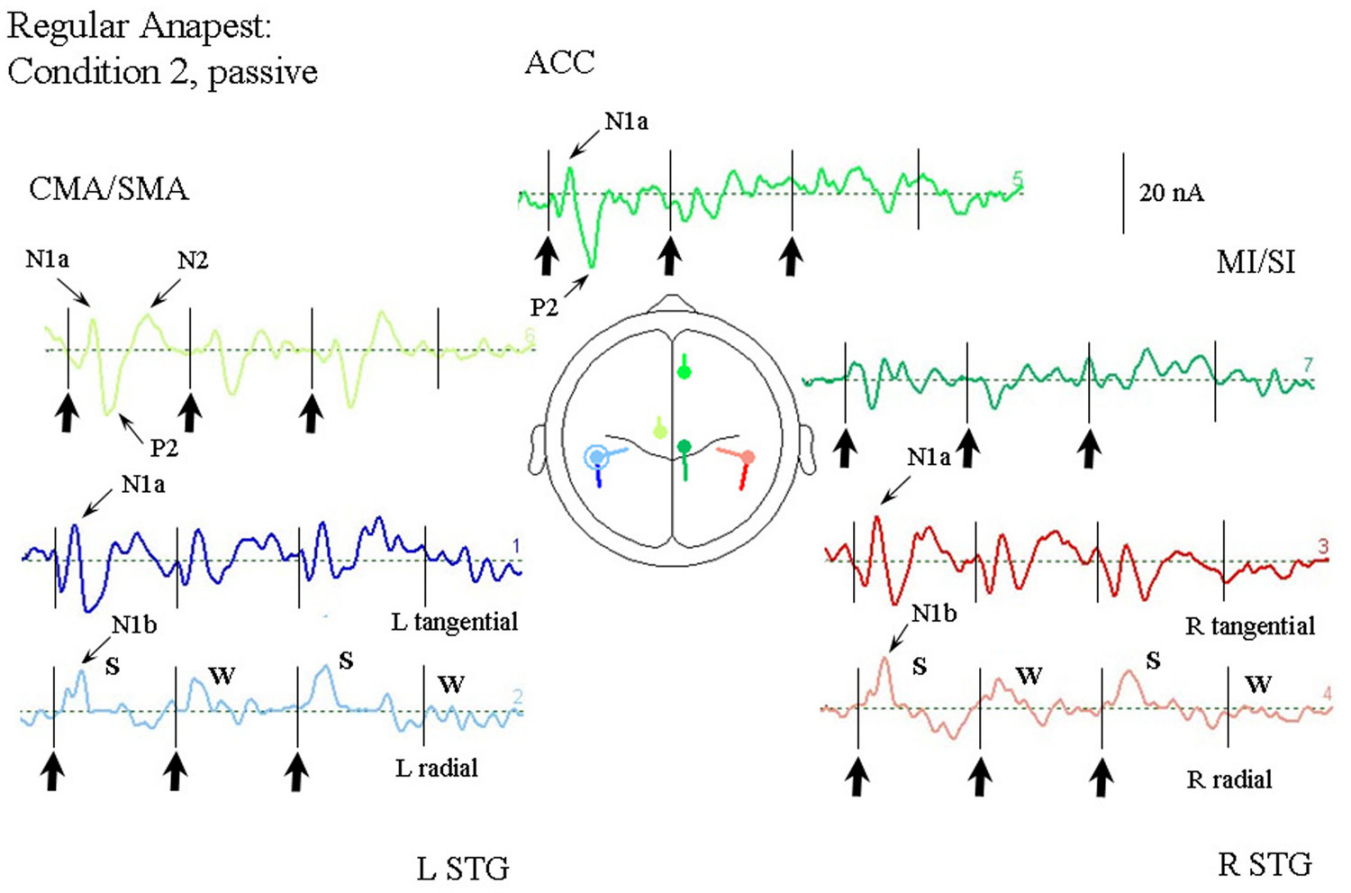
**Response of the optimum source model sources for passive listening to the regular anapest rhythm (condition 2), using the same arrangement as in **Figure 4**.** Beat onsets are indicated by vertical bars and click stimulus onsets by bold arrows. Note the appearance of a “Strong-Weak-Strong-Weak” (S-W-S-W) metrical pattern in the radial N1b source.

**Figure 5** illustrates the response of the optimum source model to the regular anapest case (condition 2). A number of features are apparent (at least qualitatively, which we test in the next section). First, overall the current values are lower than the random case (condition 1). Second the responses already appear to show evidence of metrical structure, which can be observed in particular in the rSTG sources and CMA/SMA source, indicating a S-W-S-W pattern, where S and W respectively abbreviate “strong” and “weak.” This behavior appears to be distinct from that of the tSTG sources which show more evidence of adaptation in the components. (These patterns are tested statistically in the next section).

#### Passive Listening to a Syncopated Anapest (Condition 3a)

**FIGURE 6:**
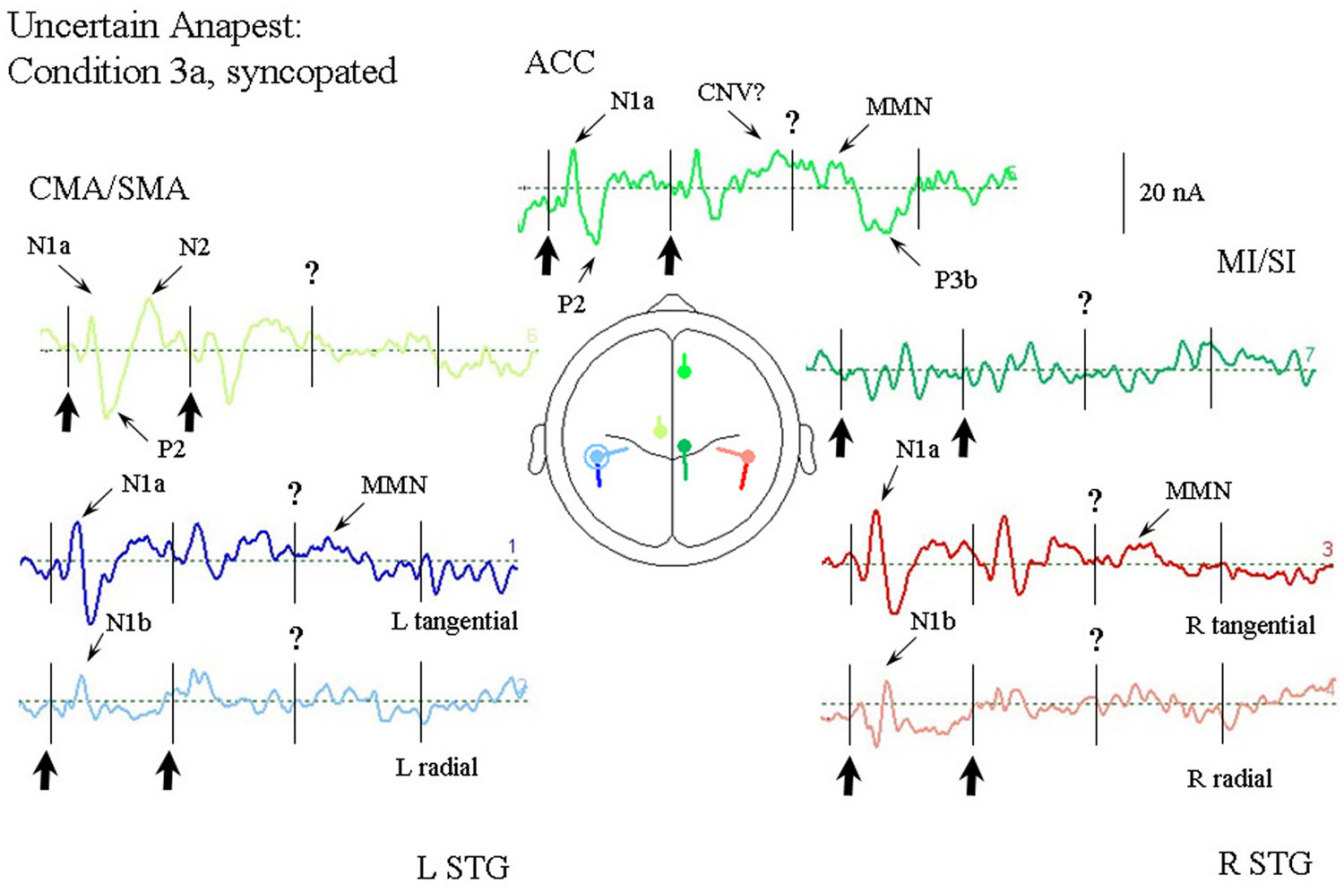
**Response of the optimum source model for passive listening to the syncopated anapest case (condition 3b).** Beat onsets are indicated by vertical bars and click stimulus onsets by bold arrows. The uncertainty over the absent third click is indicated by a question mark. The presence of uncertainty produces generally enhanced activity in the ACC source, including what looks like a CNV prior to the third beat, and a P300 (labeled P3b) following it. A miss-match negativity (MMN) can also be observed in the STGt sources and the ACC source following the omitted click.

**Figure 6** illustrates the response of the optimum source model to the uncertain anapest case with the click on the third beat omitted (condition 3a). The syncopated cycles produce responses with enhanced current magnitudes for the first two beats compared with the regular anapest, but a produce a different response for the third and fourth beats. The tSTG sources sampled at N1a on the third beat are not strongly reduced in the syncopated case (condition 3a) compared to the regular (condition 2), but when sampled at N1b are actually strongly increased by omission compared to the regular case, consistent with the MMN effect of omission. In contrast the rSTG sources sampled at N1b are reduced by omission. In addition to temporal lobe sources all frontal sources are enhanced in the syncopated condition for the first two beats, but the ACC source also shows a P3 wave (labeled P3b) following the MMN in tSTG on the third omitted beat, as well as a CNV like response prior to the third beat.

#### Passive Listening to an Anapest in the Context of a Syncopation (Condition 3b)

**FIGURE 7:**
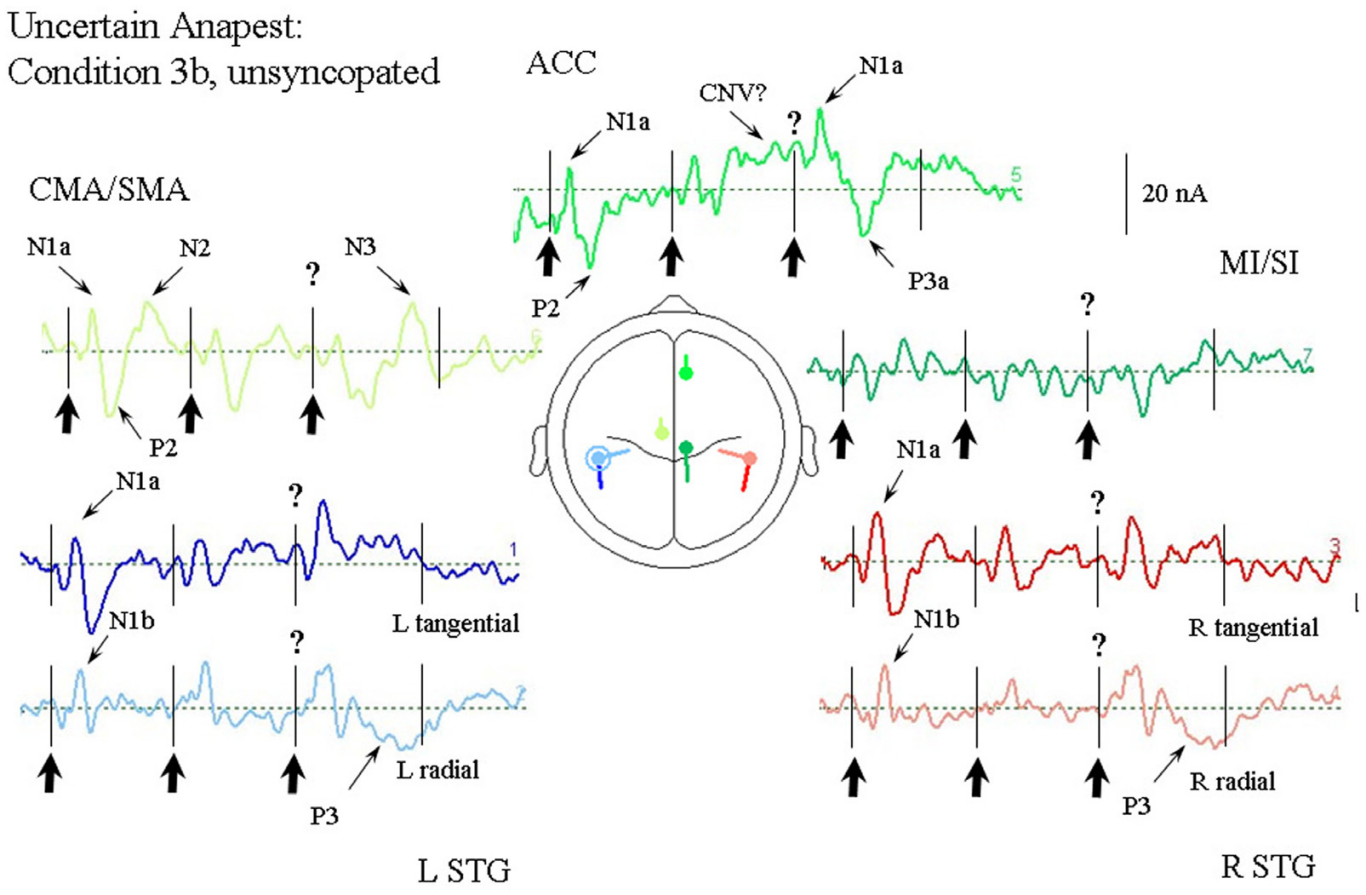
**Response of the optimum source model to the unsyncopated but uncertain anapest case, i.e., with third click present (condition 3a).** Beat onsets are indicated by vertical bars and click stimulus onsets by bold arrows. The uncertainty over the present third click is indicated by a question mark. The presence of uncertainty in this case produces generally enhanced activity in all sources. The ACC source again shows a CNV prior to the third beat, but then a large contribution to the N1a followed by an earlier P300 (labeled P3a). The STG sources all show enhanced activity at around the N1 wave. A P300 can also be seen in the STGr sources accompanied by an N300 in the CMA/SMA source.

**Figure 7** illustrates the response of the optimum source model to the uncertain anapest case with the click on the third beat present. The unsyncopated cycles (condition 3b), in common with the syncopated cycles (condition 3a), produce responses with similarly enhanced current magnitudes for the first two beats compared with the regular anapest (condition 2), as well as a CNV like response before the third beat in the ACC source, but produce dramatically different responses for the third and fourth beats. The tSTG sources sampled at N1a on the third beat are doubled in size in the unsyncopated case (condition 3b). In contrast the rSTG sources sampled at N1b are only slightly enhanced by inclusion. In addition to temporal lobe sources all frontal sources are enhanced, but with the largest change occurring for the ACC source sampled at N1a on the third beat, where the current is more than three times that for the regular anapest (condition 2), followed by a P3 wave (labeled P3a). Unlike the syncopated case, however, the P3 activity is more widely distributed and can be observed in the rSTG sources, accompanied by a following “N3” in the CMA/SMA source. Todd and Seiss (2004) reported a source analysis of the N3 wave suggesting an inferior parietal lobule (IPL) input. Given the proximity of the STG sources here to IPL then, an input from this area to the observed rSTG P3 activity is likely.

#### Synchronization with the Regular Anapest (Condition 4)

**FIGURE 8:**
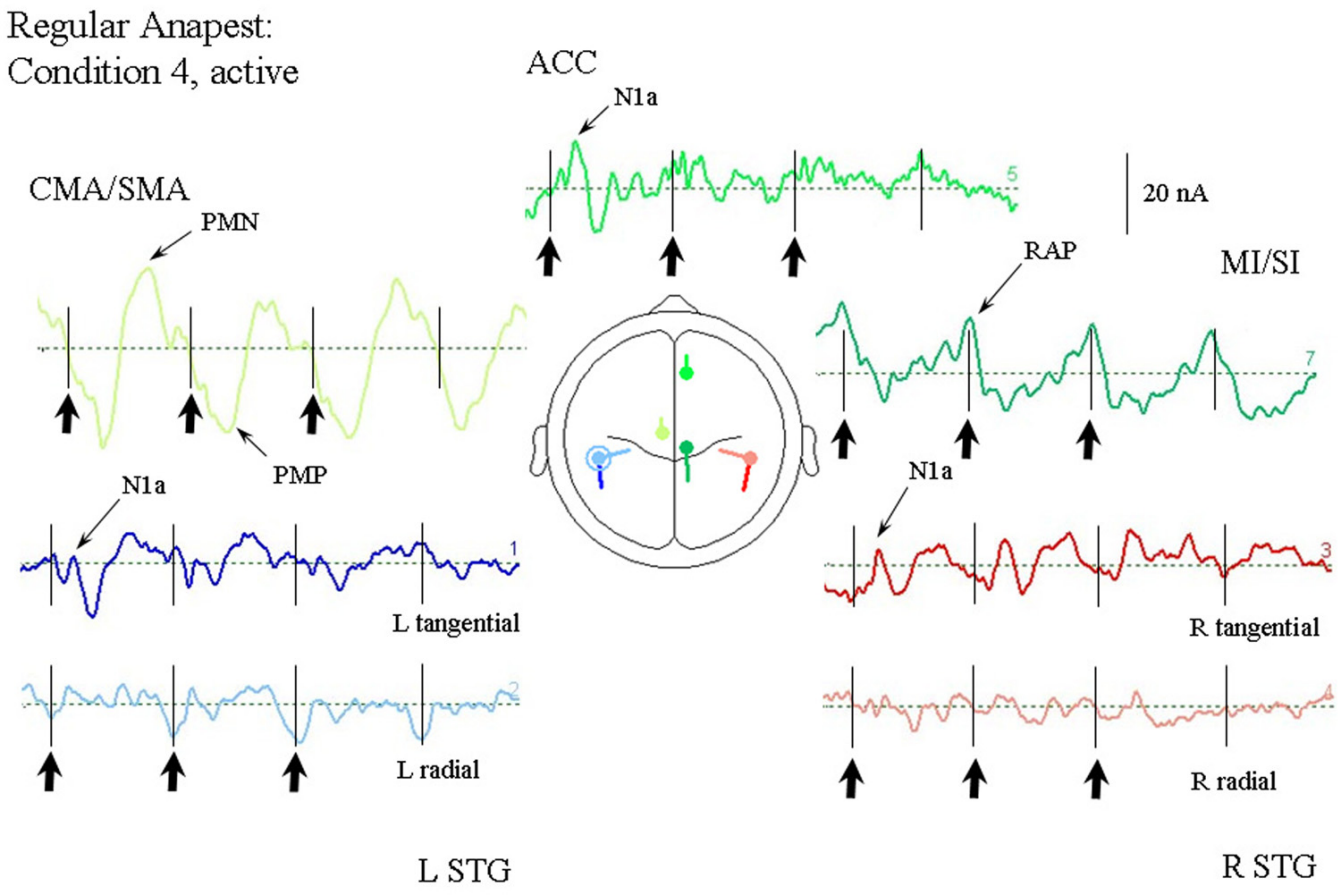
**Response of the optimum source model to the regular anapest case during active synchronization (condition 4).** Beat onsets are indicated by vertical bars and click stimulus onsets by bold arrows. CMA/SMA and MI/SI sources show enhanced and regular activity aligned with the beat cycle. Peaks corresponding to the pre- and post-movement potentials can be observed within the CMA/SMA source. Peaks aligned approximately with the beat onset corresponding to the RAPs can be observed within the MI/SI source.

**FIGURE 9:**
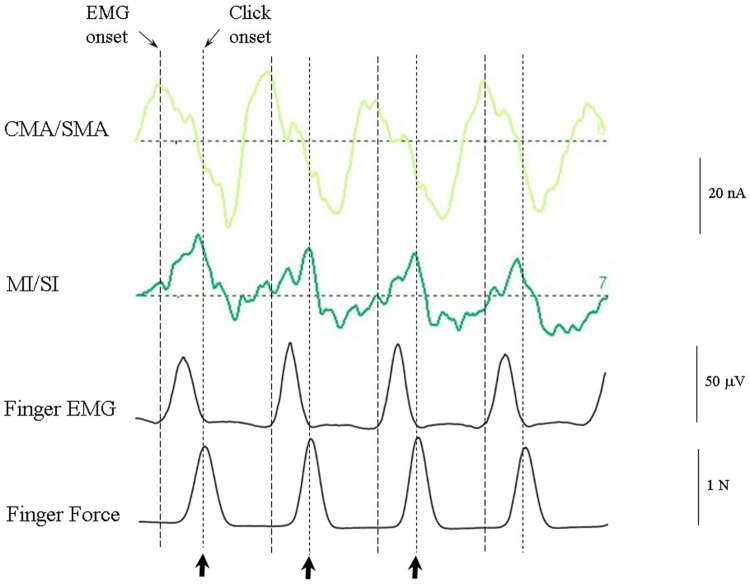
**CMA/SMA and MI/SI sources aligned with the finger EMG and finger force activity.** The CMA/SMA activity is at maximum negative (corresponding to the peak pre-movement negative wave, or PMN) at the onset of negative going MI/SI activity and finger EMG. The M1/SI activity is at maximum negative (corresponding to the peak RAP) approximately at the time of peak force exerted by the index finger on the force plate.

**Figure 8** shows the response of the optimum source model to the case where the subjects were synchronized with the regular anapest (condition 4). Both the CMA/SMA and M1/S1 sources show a cyclical pattern consistent with motor activity during synchronization. The interpretation of the cyclical patterns becomes clear, however, when aligned with the finger EMG and force activity, as shown in **Figure 9**. There were some differences in the ability of the subjects to accurately carry out the flick extension task, i.e., to only employ the extensor indices muscle, which we discussed in Todd and Seiss (2004), but the mean evoked responses for these subjects did not differ greatly from the grand mean of all subjects, so in the present analyses we included all subjects. The deeper CMA/SMA source starts becoming negative well before the finger EMG activity, but reaches its peak just about at the onset of the finger EMG. The MI/SI source starts becoming negative at about the onset of the finger EMG and reaches a peak just after the peak in force which coincides with beat onset (more precisely about 10 ms after). Given its sagittal orientation it is likely that this source is also capturing some activity in somatosensory cortex (SI) associated with the RAP, as well as motor cortex activity. This view is supported by the fact that there is a double peak in the MI/SI source, the first smaller peak likely corresponding to the motor drive, and the second larger peak to the sensory consequence of the movement. The CMA/SMA source also shows a double peak, the first larger peak likely corresponding to the movement preparation, and the second to the movement termination. For these reasons we labeled the prominent negativity in the CMA/SMA source prior to the beat onset the source of the pre-movement negative (PMN) wave and the prominent positivity after the beat onset the source of post-movement positive (PMP) wave. The peak in the MI/SI source coincident with the beat onset was labeled the source of the RAP.

#### Comparison of Passive and Active Cases (Conditions 2 and 3 vs. 4 and 5)

**FIGURE 10:**
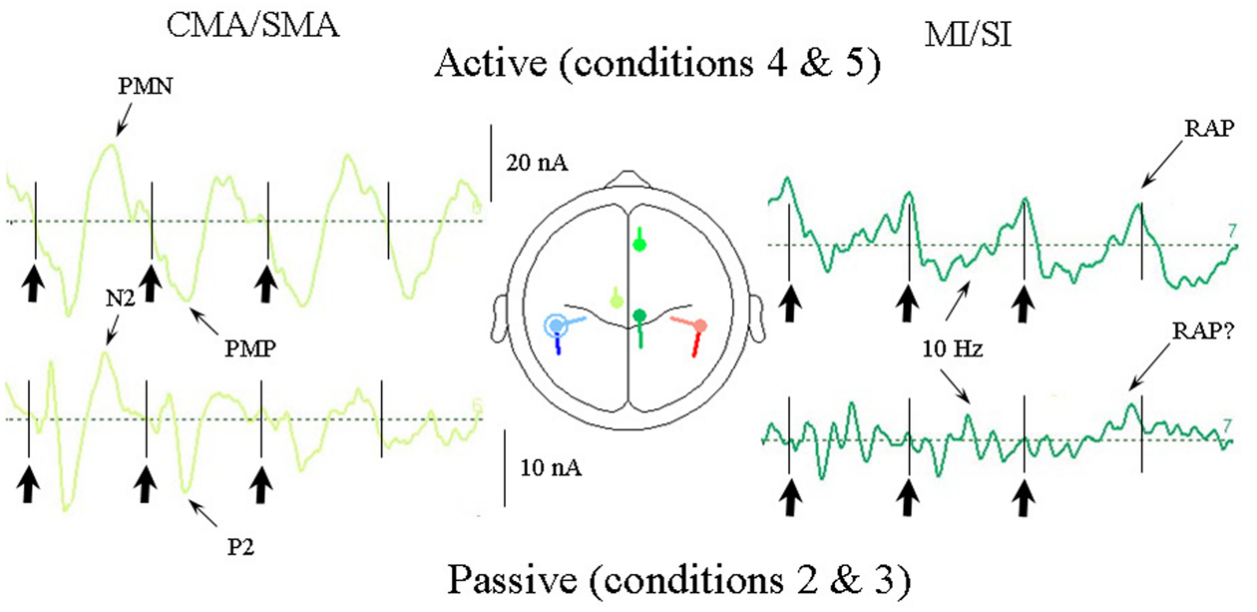
**Comparison of CMA/SMA and MI/SI source currents for the active vs. passive cases averaged over all the syncopation conditions.** For the passive cases these are conditions 2, 3a, and 3b and for the active cases conditions 4, 5a, and 5b. The CMA/SMA activity is quite closely correlated for active and passive cases indicating an association between the passive auditory P2/N2 waves and the movement-related PMN/PMP waves. Thus evidence of movement relatedness can be seen in the passive case, as well as the active case.

As the response pattern during active listening to the uncertain conditions (conditions 5a and 5b) did not differ greatly from the regular anapest condition (condition 4) we show in **Figure 10** the current source response to the average of the regular and uncertain synchronization cases (conditions 4 and 5) compared with the response to the grand mean of the passive cases (conditions 2 and 3). The comparison is limited to the CMA/SMA and MI/SI sources as these are critical to the interpretation. There are a number of features of interest in this comparison. One is that there is a strong temporal alignment of the CMA/SMA contribution in the passive and active cases, with an association between the P2 and the PMP waves and with the N2 waves and the PMN, although with the magnitude correspondingly greater in the active case. Having demonstrated above that the CMA/SMA source is plausibly associated with movement preparation in the active condition, this temporal alignment is consistent with the CMA/SMA activity in the passive condition sharing movement relatedness, as was argued by Todd and Seiss (2004).

A second feature is that within the MI/SI source there is an ∼10 Hz oscillation clearly observable in the active condition superimposed on the 2 Hz beat rate in the form of five clear cycles per beat, especially for the first and second beat periods, but also present in the third beat period. This apparent 10 Hz component can also be seen in the passive cases. In addition, of particular note is the presence of what appears to be a RAP source in the passive case on the fourth (silent) beat in the same location as that for the active case. We return to this issue in the discussion.

### Quantitative Testing of the Results

In Section “Results of the Source Analysis” above the source analysis results were presented in a rather descriptive manner. In order to test these qualitative observations in this section we report a statistical analysis on the magnitudes of current sources measured at fixed points in time across conditions for comparison after averaging across subjects. The sampling points following the stimulus correspond approximately to the long latency potentials N1a, N1b, P2, N2a, N2b, and N400 and the time points for measurement were set respectively to 95, 120, 155, 260, 300, and 390 ms after each beat onset. Although there was some variation in the latencies between conditions, the use of a fixed set of latencies allowed an accurate comparison of sampling the sources in time across conditions, an exception being for the random condition (condition 1) where the N1b and P2 were measured at 130 and 180 ms. In the original measurement configuration the seven current sources were treated as subjects, and the six sampling points per beat as within-subjects factors (e.g., as in **Table 2**). Thus for each of the seven sources there were 80 samples each for the active and passive cases. It was, however, convenient to carry out the analysis on a transposed version of the data array so that the current sources were treated as within-subjects factors and the current sampling points treated as subjects, separately for each of the four beats and each of the rhythmic conditions. Beat position, rhythmic condition and wave number could then be treated statistically as between-subjects factors. For the present analysis the data structure was simplified by summing the seven current sources into two levels of a within-subjects factor of “Lobe,” with the two levels labeled “Temporal” (i.e., the sum of L tSTG, L rSTG, R tSTG, and L rSTG) and “Frontal” (i.e., the sum of ACC, CMA/SMA, and MI/SI).

Considering first the overall effect in an ANOVA with lobe (i.e., temporal, frontal) as a within-subjects factor, and with rhythmic condition [i.e., irregular (condition 1), regular (condition 2), uncertain regular (condition 3b), uncertain syncopated (condition 3a)] and wave (i.e., N1a, N1b, P2, N2a, N2b, and N400) as between-subjects factors for the passive cases, there were main effects of rhythmic condition, *F*(3,54) = 12.0, *p* < 0.001, and also of wave, *F*(5,54) = 6.8, *p* < 0.001, and of lobe, *F*(1,54) = 5.0, *p* < 0.05. These effects confirm the qualitative impression that the magnitude of the currents varies considerably between rhythmic conditions such that the current activity during the irregular condition (condition 1) was larger than the regular (condition 2), but with the uncertain conditions (conditions 3a and 3b) somewhere between. Overall the P2 wave was the largest and frontal lobe currents slightly larger than temporal lobe currents.

**FIGURE 11:**
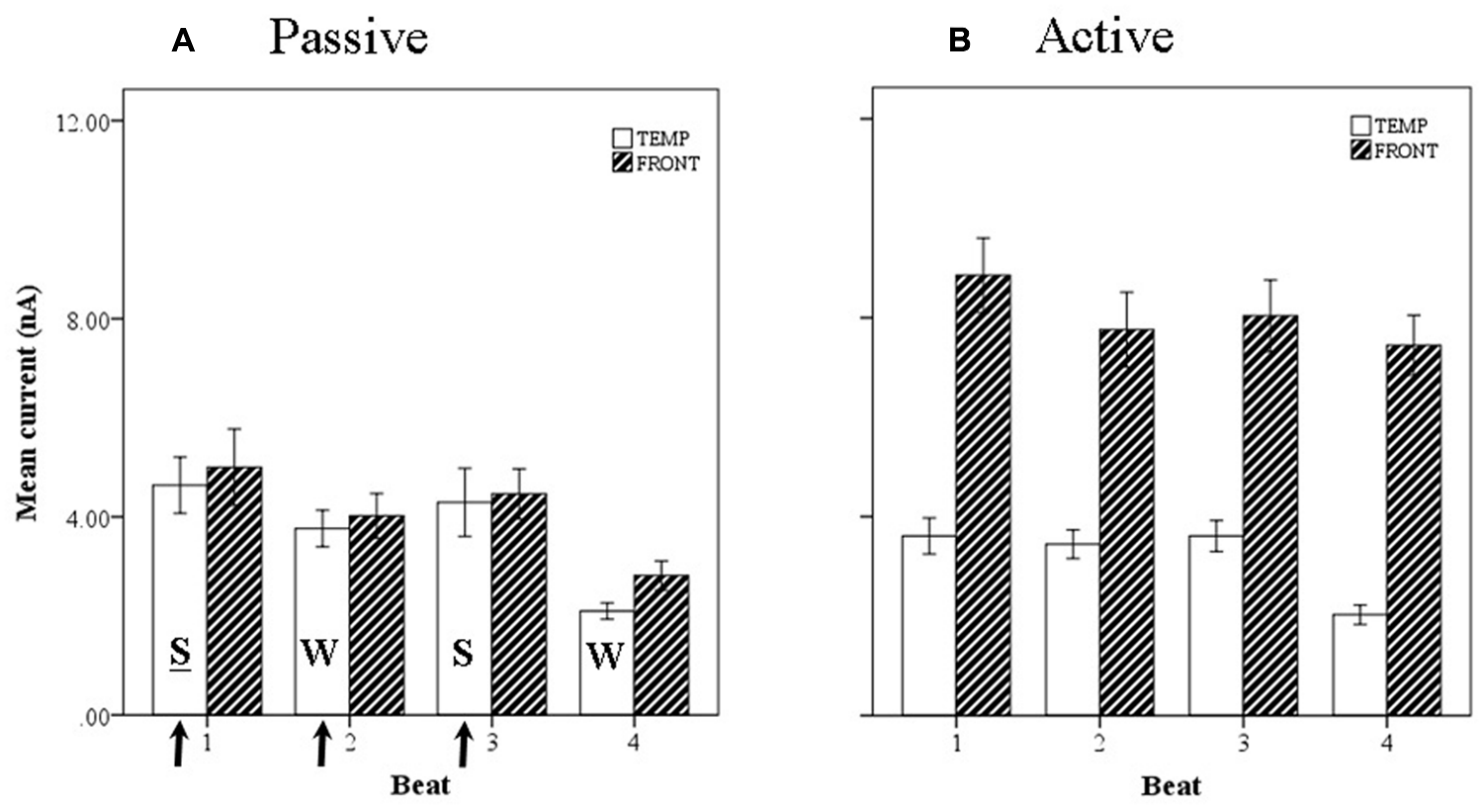
**Marginal means of source currents showing the effect of beat position for the mean of frontal and temporal lobe sources during **(A)** passive listening and **(B)** active synchronization.** Error bars show standard error. The arrows indicate the alignment of the click stimulus. In the passive conditions the current activity on beats 1 and 3 is relatively larger than on beats 2 and 4, indicated by the labels S-W-S-W.

**FIGURE 12:**
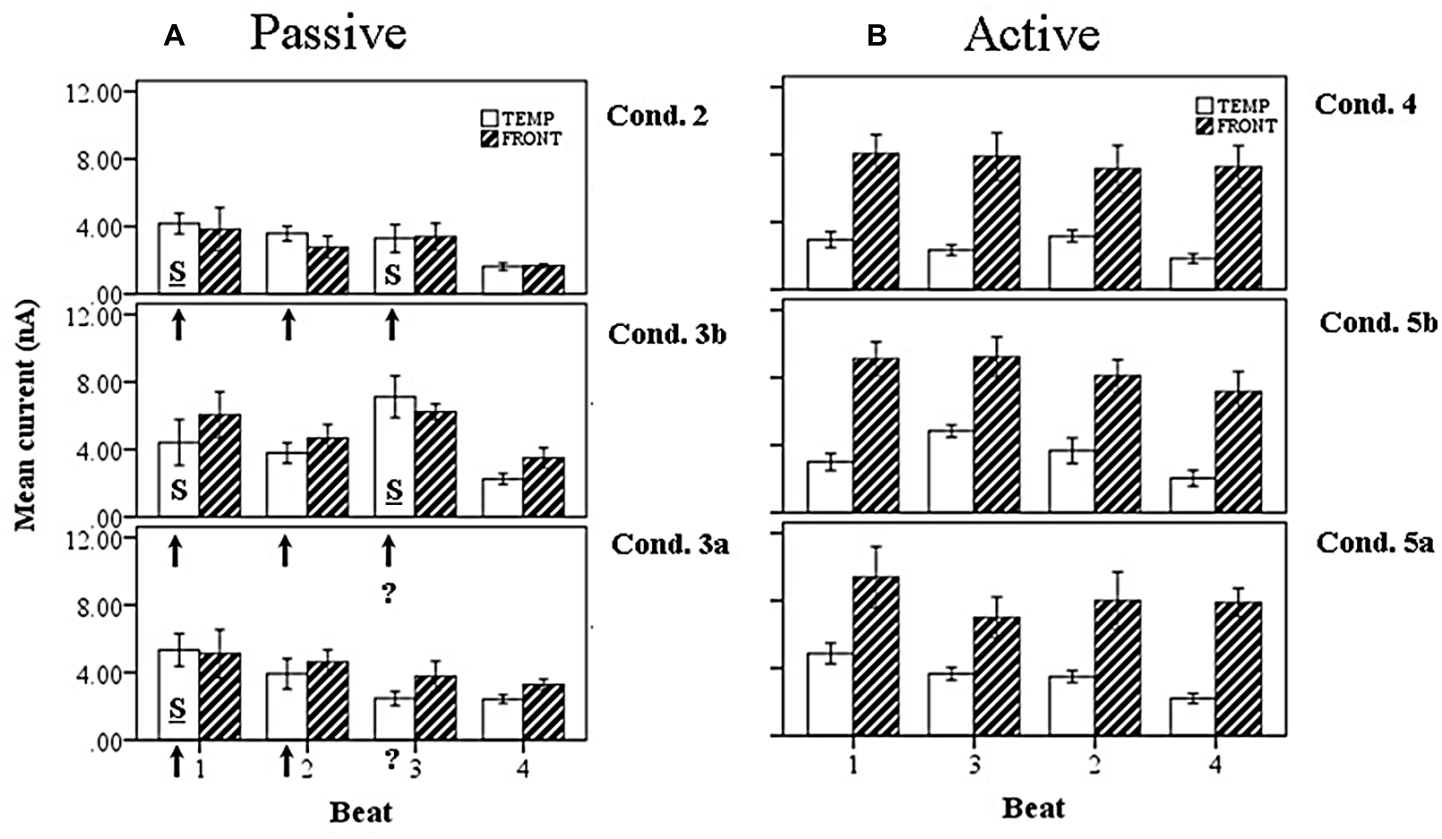
**Marginal means of source currents showing the effect of beat position for the mean of frontal and temporal lobe sources, broken down by syncopation condition, during **(A)** passive listening and **(B)** active synchronization. Error bars show SE.** The arrows indicate the alignment of the click stimulus, with the “?” symbol used to indicate uncertainty on the third beat. In the passive cases the effect of uncertainty is to enhance activity on the third beat when the click is present (condition 3b) and shift the stress pattern to S-W-S-W. For the syncopated case (condition 3a) the activity on the third beat is reduced, resulting in a S-W-W-W pattern.

Introducing now the effect of beat position for the three anapest rhythm conditions [i.e., regular (condition 2), uncertain regular (condition 3b), uncertain syncopated (condition 3a)], the overall effect of lobe and beat position on current marginal means (means for that factor averaged across all levels of the other factors) are illustrated for passive and active cases in respectively **Figures 11A,B**, and of rhythmic condition in **Figures 12A,B**. For the passive conditions (**Figures 11A** and **12A**) there is no overall effect of lobe, but significant main effects of beat, *F*(3,60) = 7.3, *p* < 0.001, and rhythm condition, *F*(2,60) = 6.8, *p* < 0.005. Thus we see that a metrical structure is evident across beat position with alternating “strong” (S) and “weak” (W) beats. This pattern is present independently present in both temporal and frontal sources (**Figure 11A**) and is enhanced in the context of syncopation for the case when then the third beat is present (condition 3b) (**Figure 12A**). Although the interaction between beat position and rhythm condition is not significant in this sample, there is a shift in the beat pattern from S-W-S-W to S-W-S-W, i.e., from first to third beat from the regular condition (condition 2) to the uncertain regular condition (condition 3b) (**Figure 12A**), as noted in the qualitative description above. When applied to the active conditions (**Figures 11B** and **12B**) the effect of lobe becomes highly significant, *F*(1,60) = 184, *p* < 0.001, where the frontal lobe contribution is much larger then that of the temporal lobe, but the effects of beat and rhythm condition are abolished. Although the metrical pattern is still discernable, it does not result in a significant beat effect in this sample.

**FIGURE 13:**
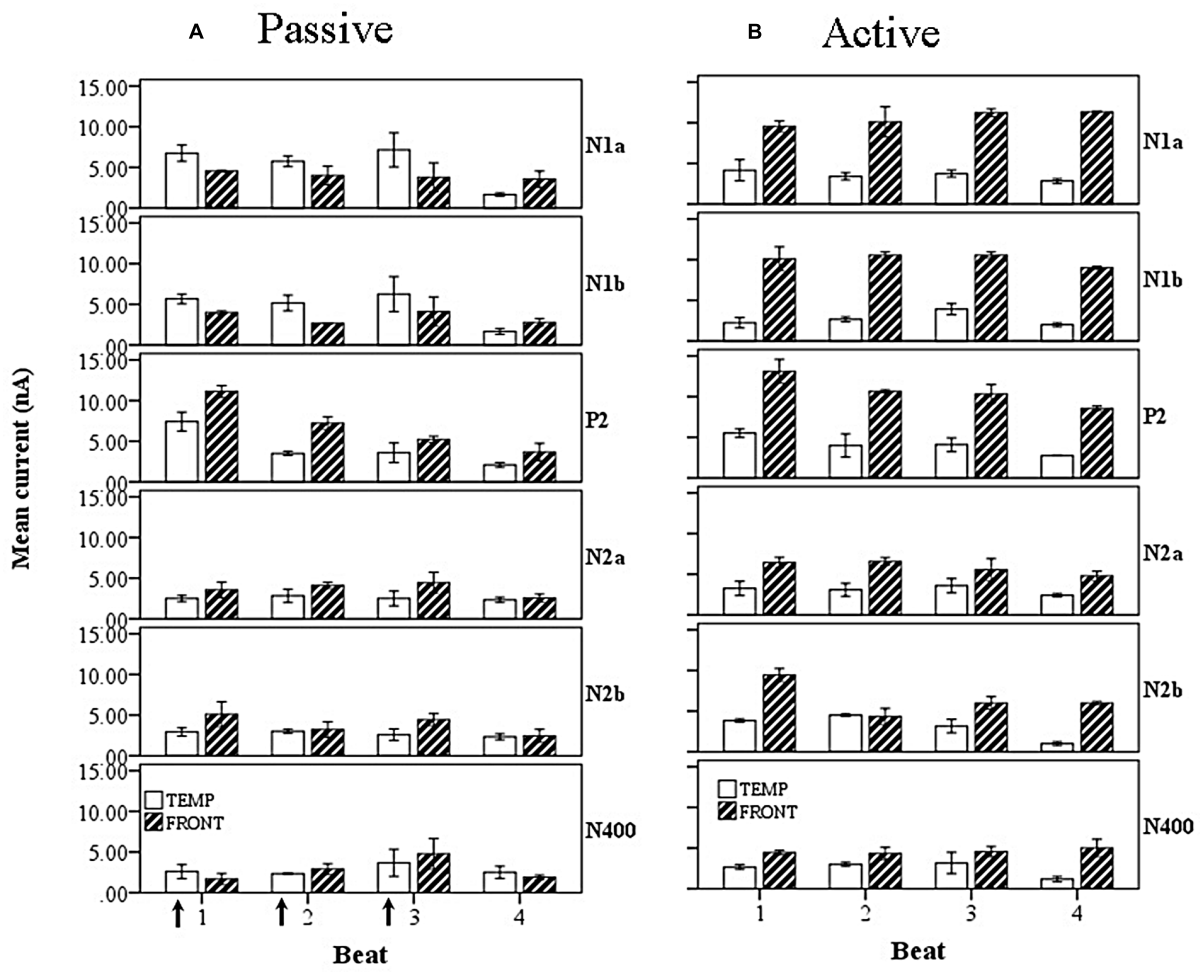
**Marginal means of source currents showing the effect of beat position for the mean of frontal and temporal lobe sources, broken down by wave, during **(A)** passive listening and **(B)** active synchronization.** Error bars show SE. The arrows indicate the alignment of the click stimulus. In the passive case both components of the N1 show greater temporal than frontal lobe activity and both exhibit a S-W-S-W pattern of current activity. The later P2 and N2 components show larger contributions from the frontal lobe sources.

When the wave factor is introduced for the passive rhythm conditions, illustrated in **Figure 13A**, a main effect of wave is observed, *F*(5,48) = 6.4, *p* < 0.001, as well as a two way interaction between wave and lobe, *F*(5,48) = 8.8, *p* < 0.001. Thus, as noted above for the overall comparison, the P2 is the largest wave, and the interaction shows, as is clear from **Figure 13A**, that the early waves, i.e., N1, have a larger temporal cortex input, whereas the later waves are more frontal. For the active rhythm conditions (**Figure 13B**), once again a very large effect of lobe is seen, *F*(1,60) = 544, *p* < 0.001, and again a main effect of wave, *F*(5,48) = 28, *p* < 0.001, but also an interaction of wave and lobe, *F*(5,22) = 8.8, *p* < 0.001. In this case the interaction indicates that the frontal sources contribute more to the early part of the beat period, generated from the PMP which aligns with the P2.

### Discussion of the Results

We showed first that a source model implicating bilateral temporal lobe and three frontal areas gives a good description of potentials produced by the irregular stimulus (condition 1). The validity of the model is judged both by the goodness of fit (2.6% residual variance), its agreement with other independent modeling cases, and the degree to which the sources locate to areas previously indicated by other methods, including fMRI. On all three these counts the model is a good one, we believe. We then applied the model without change (apart from switching off the second tangential sources for clarity) to all the other rhythm conditions in the experiment, including the active conditions. The interpretation of the three frontal sources was given support, and the model further validated, by the fact that their activity was dissociated, especially in the active rhythm conditions, i.e., the CMA/SMA and MI/SI sources could be clearly interpreted, by reference to the EMG and force profile, as contributing respectively to the pre-/post-movement potentials (i.e., PMN/PMP) and motor/reafference potentials.

The first result from the above analyses is that metrical structure in the form of a pattern of alternating relatively strong and weak currents can be identified which is consistent with our intuitions about the metrical structure associated with the anapest, i.e., that the first and third events should map onto strong beats. This result we believe is a novel one. Although metrical structure is implied by P300 and MMN studies from omission, as reviewed in Section “Auditory Evoked Potentials (AEPs),”, and some evidence of metrical structure has been presented in beta and gamma band oscillation studies, as reviewed in Section “Movement/Stimulus Preceding Potentials,” the direct observation of metrical patterns in current sources in response to regular, non-accented rhythmic patterns without omission, has not been previously observed in an EEG study. Some evidence has been presented for metricality from frequency analysis of EEG and direct temporal lobe studies (Nozaradan et al., 2011), and this result is consistent with this evidence. However, the result here that this can be observed in both temporal and frontal sources on a beat by beat basis has not been achieved previously.

The second result is that both temporal and frontal sources contribute to all waves, and this we suggest provides new evidence consistent with the theory that the experience of metrical structure is a sensory-motor phenomenon. In addition, as well as being distributed between sensory and motor sources, the metricality is distributed in time between beat onsets, contrary to most claims made on the basis of the beta/gamma band studies which generally locate metrical activity close (within about 100 ms) to the beat onset (see Movement/Stimulus Preceding Potentials). The “beta rebound” observed by Fujioka et al. (2012) is perhaps one exception. Thus, as well as being distributed throughout the sensory-motor network this current activity is distributed in time over the course of the rhythm.

A third result here is that the introduction of syncopation increases the total current activity for both uncertain conditions, i.e., when the third click is present (condition 3b) and omitted (condition 3a), compared to a regular rhythm (condition 2). This result was also robust, and again is independently obtained for both temporal and frontal lobe source components. Of particular importance was the fact that when the third click was present in the context of syncopation (condition 3b) the current activity was greatly increased on the beat where the syncopation had occurred, i.e., beat 3. This caused a shift in the pattern of current activity so that although the S-W-S-W pattern was preserved the emphasis shifted from the first beat to the third beat.

Again, although effects of omission on P300 and MMN, and increases in brain activity during active motor syncopation (Mayville et al., 2002) have been observed, the direct observation of an increase in current activity in a non-syncopated rhythm in the context of a prior syncopation we also believe is novel. Within the frontal sources the ACC sources was in particular most strongly modulated by a syncopation context. Further we observed two distinct P3 activities in the context of syncopation for the two cases of when the third beat was omitted or not omitted.

As we noted above, the present source model differs from the previous approach in allowing all sources to contribute to each of the waves. However, there were some distinct effects on the relative contribution so that overall in the passive listening cases the temporal sources contributed most strongly to the N1a and N1b, with each being most strongly represented by respectively the tangential and radial temporal lobe components and the P2 and N2 waves having their largest contribution from the frontal sources. This and the fact that the there was quite a strong temporal correlation between the pre-/post-movements potentials and the P2/N2 waves provides further support for the view expressed in Todd and Seiss (2004) that when the stimulus rate matches to regular body motions, frontal, i.e., CMA/SMA, contributions to the N2 may become entrained as PMNs, even if there is no significant activation of MI.

In Todd and Seiss (2004) we argued that the apparent overlap of the N2 and the PMN had its likely origin in the N2 being a manifestation of an “orientation reflex” (Näätänen and Gaillard, 1983). The fact, however, that motor areas contribute to all the AEPs means that this cannot be the sole explanation here of strong motor contributions to the passive listening cases and indeed in the irregular stimulus case. The most likely answer we suggest is that the stimulus used here was above the vestibular threshold, so that vestibular projections to the cingulate cortex were activated by sound. As we noted in Section “Source Analysis of Electrophysiological Correlates of Beat Induction,” and in the discussion of the motivation for the reanalysis, new data strongly suggests a vestibular contribution to AEPs, and in particular to the cingulate area and to radial sources in the temporal lobe. The fact of the cingulate and prominent radial sources here is evidence that the stimulus was indeed above vestibular threshold. We return to this issue in the general discussion.

As we noted in Todd and Seiss (2004) the synchronization strategy of our subjects in this case locked in the peak of reafference to the beat onset which coincides with the force peak. In the present synchronization data and source analyses we see that the negative peak in the MI/SI source aligns with the beat onset, which is preceded by a smaller motor potential and a larger readiness potential in the CMA/SMA source. As noted above the CMA/SMA waveform appeared to match up with the P2 and N2 waveforms. This raises an issue of how the temporal and frontal sources might be linked. The appearance of an ∼10 Hz oscillatory component most prominent in the MI/SI suggests a possible role in sensory-motor coupling during synchronization (Erimaki and Chrustakos, 2008; Castro-Alamancos, 2013).

## General Discussion

We have in the above reanalysis provided some new evidence which is relevant to the sensory-motor theory of rhythm and beat induction. The main finding is that the metrical structure is manifest in greater current activity for strong compared to weak beats of the anapest. In addition the outcomes of modeling suggest that: (1) activity in both temporal and frontal areas contribute to the metrical percept and that this activity is distributed over time, i.e., not localized to beat onsets; (2) activity is greatly increased when a temporal expectation is confirmed following a previous violation, such as a syncopation; (3) two distinct processes are involved in auditory cortex, corresponding to tangential and radial (possibly vestibular dependent) components of the temporal lobe current sources. In the rest of this general discussion we consider the wider implications of these results.

### What is the Origin of Binary Bias of Metrical Structure?

One of the main findings of the vestibular based model was that there was a natural tendency for a binary S-W-S-W structure in the current activity (e.g., see **Figure 11A**), consistent with much prior literature on the effects of position in sequence on perceived accentuation (e.g., Povel and Okkerman, 1981; Brochard et al., 2003). This can be explained in a number of ways. One possible explanation is the existence of distinct processes of adaptation and integration (Loveless et al., 1996), the first of which makes the first event of a sequence the strongest, the second of which places stress on the last event of a sequence. Evidence of both can be found in our data in detailed analyses in both the temporal and frontal areas. The combination of such processes could produce the binary current pattern that we observed. Such processes would be examples of factors contributing to what is termed a phenomenal accent (Lerdahl and Jackendoff, 1983; Todd, 1994), in this case stress caused by the position of the event in the sequence.

Another possible explanation is that RFs in auditory cortex possess temporal band-pass filter properties tuned to a range of modulation frequencies, as reviewed in Section “From Sensory-Motor Theoretic Constructs to Observed Sources,” which phase lock to events and give rise to a power spectrum. Collectively the output would tend to emphasize events associated with the onsets of periods at different levels, and thus give rise to greater current activity on those events. Unlike the adaption/integration mechanism, such a spectral mechanism would have a binary bias because of the harmonic series: the first metrical harmonic would always be double the fundamental. There are in existence several models which make use of wavelet type filter mechanisms, which can be compared to cortical RFs, in order to compute a continuous spectral metrical grid-like representation (e.g., Todd and Brown, 1996; Todd et al., 1999, 2002; Cemgil et al., 2000; Smith, 2000; Smith and Honing, 2008; Tomic and Janata, 2008). Although there is some variability between such spectral models, they will likely yield the same binary bias effect due to the harmonic series being represented.

A third explanation which arises from the sensory-motor theory is that the particular motions evoked by a regular rhythm are likely to be locomotor or pendular body cyclical motions (Todd, 1999; Todd et al., 1999). These were previously hypothesized because they could explain the existence region of the “tactus” (Todd and Brown, 1996). This viewpoint has subsequently received much support in the literature (e.g., Styns et al., 2007; Toiviainen et al., 2010). Such motions would also have a natural binary tendency, in contrast to the synchronization task employed here involving a single digit and a single action and muscle. Although this was essential to be able to identify with certainty the premotion and motor potentials in relation to the finger EMG and force for experimental purposes, it likely had the effect here of reducing or abolishing the metrical structure compared to the passive condition. In any real locomotor, cyclical dance or even simple head bobbing synchronization, there would be a complex set of muscles and actions involved invariably requiring paired antagonist sets of flexors and extensors and opposite, possibly anti-phase left-right alternations. Thus the pre-motion and motor potentials of such complex combinations will introduce at least a binary alternation in each of the somatotopic areas corresponding to the appropriate body parts in each of the hemispheres. Indeed any left/right asymmetry could overall produce a binary bias.

In addition to the motor alternations, there will also be vestibular sensory alternations (Phillips-Silver and Trainor, 2008; Trainor et al., 2009), depending on the particular head motion involved with the action and associated combination of vestibular receptors, which could potentially include all 10, i.e., the three canals and two otolith organs for each ear. Furthermore, otolithic receptors are organized in a morphologically polarized manner with hair-cells arranged in push–pull manner (Benson, 1982). Thus again, the direct vestibular activations will also contain binary and possibly more complex structure, which will be projected to all of the vestibular areas, including the temporal and frontal lobe areas. Since the radial temporal lobe currents in particular may be vestibular dependent (Todd et al., 2014b), they would more likely tend to possess the binary bias, and this perhaps explains the differences we observed here between the tangential and radial temporal lobe sources.

One additional factor must be thrown into the above mix, which is that it is well-established that both auditory and vestibular systems show distinct hemispheric biases, and, of course, the motor system and entire brain is asymmetrically organized. In the case of the vestibular system, there is quite a lot of evidence that it is biased to the non-dominant hemisphere, i.e., the right hemisphere for right-handers (e.g., Todd et al., 2014b). Thus even with equal inputs to the ears and a perfectly symmetrical cyclical motion, the two hemispheres will not represent the patterns equally, and this will introduce a bias into the metrical interpretation. Thus we may see that a combination of peripheral accenting effects, metrical harmonic series effects from auditory RFs, effects of vestibular and motor patterning that goes with cyclical motions, as well as hemispherical asymmetries, will all combine to produce binary or more complex interactions.

### Why Does Syncopation Make you Move and Why is it Pleasurable?

The second main finding was that confirmation of a temporal expectation, following a previous violation in the form of syncopation, gives rise to an increase in current activity (e.g., see **Figure 12A**). We suggest that this may provide at least part of the answer to the question as to what makes syncopation effective. The factors which give rise to syncopation and the strength of both the compulsion to move and “pleasure” which is associated with it are now well-established. According to Witek et al. (2014) there is an inverted U-shaped empirical relationship between syncopation, body movement and pleasure. However, although expectation-based accounts have been proposed as to why syncopation is pleasurable (e.g., Huron, 2006), there is presently no accepted explanation of why syncopation gives rise to a compulsion to move and why such movement is pleasurable. We believe that the model presented here in the context of the new synthesis provides at least the beginning of such an account for the following reasons: (1) activity in the frontal sources is increased by syncopation, (2) the frontal sources are vestibular dependent and (3) vestibular activation is naturally rewarding.

The starting point is the observation here that in addition to temporal lobe sources, frontal sources, and in particular CMA/SMA and ACC sources, are activated when listening passively to a rhythm, consistent with observations in the literature (Schubotz, 2007; Zatorre et al., 2007; Grahn and Brett, 2007; Chen et al., 2008). These areas are activated even in an irregular temporal sequence, but activity in these areas both becomes increased in syncopated rhythms compared to regular unsyncopated rhythms and to the irregular cases. In particular the ACC sources are greatly enhanced when a beat is confirmed in the context of a prior syncopation. This observation we suggest underlies the pleasure aspect of syncopation because the ACC is associated with reward (Koelsch, 2014). The activity in the CMA/SMA source takes on the form of pre-movement/post-movement potentials during a regular rhythm, but these pre-movement/post-movement waveforms are also increased during syncopation. We suggest that this in conjunction with the ACC activity underlies the compulsion to move. Thus both pleasure and wanting to move can be explained by the combined increase of ACC and CMA/SMA activity due to syncopation.

Anatomically these two regions are distinguished by the rostral and caudal subdivision of the CMA, i.e., so that ACC corresponds to the rostral CMA and the CMA/SMA source the caudal CMA (Todd and Lee, 2015), and functionally these two areas are distinguished by their connectivity and role (He et al., 1995; Hatanaka et al., 2003; Takada et al., 2004; Arienzo et al., 2006). The rostral CMA is most strongly connected to pre-frontal cortex, underlying its role in making reward based selection of voluntary movement. The caudal CMA with its proximity to M1 and SMA has a stronger role in the direct control of movement. Both rostral and caudal CMA are somatotopically organized, as are the SMA and M1, and both receive strong inputs from the vestibular system. It is this vestibular connectivity to the cingulate region in particular, we suggest, that underlies the pleasure aspect of syncopation. As described in Todd and Lee (2015), vestibular reward, which is innate (Phillips-Silver and Trainor, 2008; Trainor et al., 2009), from an early age produces what we termed the “dance habit” which is a learned association between a musical beat and the reward that is obtained from an actual motion. The learned habitual motions most likely to be selected during beat induction are stereotyped head movements, such as head bobbing or nodding. Once the head is actually moving then the vestibular reward becomes more direct, but, as we noted above, given the acoustic sensitivity of the vestibular system, a direct vestibular input can be obtained from low-frequency sound or vibration. This also explains why it is that when music has got a groove there is a tendency to turn the volume up, so that the intensity of vestibular activation and hence reward is increased.

In the Section “Discussion of Results” we considered the relationship between the P2/N2 and PMP/PMN potentials during a rhythm where the stimulus rate corresponded to natural beat rates and noted that this might have its origin in the N2 being a manifestation of an “orientating reflex” (Näätänen and Gaillard, 1983; Todd and Seiss, 2004). In Todd and Lee (2015) we expanded on this idea that the N2 represents a readiness for action cognitive reflex which may become entrained to form a pre-movement negativity. The new data by Todd et al. (2014a,b) and the results here provides strong evidence that the N2/PMN “orienting reflex” link may be vestibular dependent because both are generated in cingulate cortex, or at least include a component generated in cingulate cortex. It makes sense that the orienting reflex should be vestibular dependent because the vestibular system is central to a number of reflexes. The classical vestibular reflexes involving the vestibular-spinal and vestibular-ocular pathways are well described. Less well described, but nevertheless established, is what is referred to as a vestibular-autonomic reflex. This is mediated by a sub-cortical and cortical network which can produce autonomic responses (Balaban, 2002; Todd and Lee, 2015). The cortical components of the network feature the areas associated with PMN, readiness potentials and the N2 we suggest.

In normal function the vestibular system plays a critical role in controlling and modulating all of the reflexes for maintaining posture, gaze and autonomic function, especially if there is a perturbation, such as stumble during locomotion, and this we suggest may be the origin of its activation in syncopation. As we noted above, a critical part of the sensory-motor theory of beat induction is that the kinds of motions evoked by beat induction are locomotory, so that we could think of a syncopation as being a bit like a trigger for a reflex response to a perturbation or stumble during locomotion, followed by relief or reward if the cue is confirmed as a false alarm.

### What are the Implications for Oscillator Theories of Beat Induction?

It has been suggested that linear filter models of the kind proposed within the framework of the sensory-motor theory are essentially a kind of oscillator model (Large, 2008), akin to the adaptive non-linear oscillator models originally proposed by Large and Kolen (1994), McAuley (1995, unpublished PhD Thesis), and Large and Jones (1999). Such models have become the established explanation of the origin of beat induction and many variants have subsequently been developed (for review see Repp, 2005; Large, 2008; Repp and Su, 2013). We would argue though, that a linear constant-Q or wavelet filter-bank, as in the model proposed by Todd (e.g., Todd et al., 2002 and for variants see Cemgil et al., 2000; Smith, 2000; Smith and Honing, 2008; Tomic and Janata, 2008) is fundamentally different from a non-linear bank because the impulse response of the individual filter is more localized in time and has a scaling property (see also discussion in Todd and Lee, 2015). The collective output of a bank of such linear filters constitutes a passive wavelet transform of the original signal. As we have described above, such response properties can be seen in RFs within sensory cortex and are thus plausible models of the representation of rhythmic patterns in the sensory (including auditory) areas of the brain.

In the light of this view, it is worth reflecting on the present status of oscillator theories of beat induction. According to the original concept of an “attentional oscillation” (Large and Kolen, 1994; Large and Jones, 1999), which adaptive oscillator models were designed to represent, the more regular a rhythm the greater the “attentional resonance” it should generate in the “neural oscillators” (although there have been some exceptions, e.g., Large and Palmer, 2002). Apart from the fact that this generally runs contrary to the observations reproduced here, that for a regular rhythm the neural activity is less than for a syncopated rhythm or an irregular rhythm, the oscillator theory in its modern form has moved away from these older concepts and has been superseded by proposed beta and gamma oscillatory processes of much higher frequency, i.e., >10 Hz (Large, 2008). As we have described above in Section “Movement/Stimulus Preceding Potentials,” these processes are quite distinct from the original adaptive oscillator concept, in that their proposed function is primarily is one of cortico-cortical binding of sensory and motor areas. While we are in agreement that some oscillatory processes may indeed be associated with the consequences of beat induction, via the periodic binding of sensory and motor areas, these do not in themselves directly represent the beat process itself. We suggest that this raises a particular challenge to oscillator accounts of beat induction because the consequence is that the new beta/gamma band based oscillation theories can no longer offer an explanation of beat induction. We cannot say that humans experience beat induction because there exists in the brain a network of oscillators, only that when humans do experience a beat we can observe an oscillatory signal of binding that is a result of beat induction.

## Conclusion

In the general discussion above we have expanded on the two principal findings that emerged from the reanalysis of the Todd and Seiss (2004) data, i.e., (1) that metrical structure emerges from the current sources analysis when the activities are sampled over the whole beat period and in distributed temporal and frontal areas, and (2) that the analysis shows appropriate sensitivity to metrical surprise and may therefore go some way to explain syncopation. As well as being novel results in themselves, we believe for a number of reasons that these also suggest a new methodological approach. First, in contrast to the beat following or beat predicting electrophysiological studies reviewed above, the present approach is an integrated combination of the two, so that, for example, the N2 here is treated as both a stimulus following potential and a pre-movement potential. By conducting the analysis jointly in terms of both temporal and frontal generators there is no real distinction between following and predicting, only that the balance of auditory vs. motor in the mix may vary. Second, the present analysis does not require unusual stimulus probabilities to be able to have access to mechanisms of beat induction and syncopation. Metrical structure emerges and can be measured directly even for a regular rhythm for which there is no variability or perturbation. This stands in contrast to the MMN or P300 approaches which rely on the oddball paradigm. Third, the present analysis considers as relevant to the assessment of a beat strength the total current activity of the beat cycle, and not just activities at the beat onset or just after the beat onset, as is the case for most beta/gamma oscillator studies.

A final question which might arise from the above, is whether vestibular and motor involvement is absolutely necessary for the perception of rhythm and meter (a “strong” motor hypothesis), so that there can be no perception of rhythm at all without some (pre-) motoric involvement, or whether their involvement only plays a role in modifying auditory perception (a “weak” motor hypothesis), e.g., to lead to the propensity to move or derive pleasure from rhythm. From the above data it seems clear that the brain activity associated with rhythm processing appears to be distributed in temporal and frontal sources, but the balance between the two varies over time in a continuous manner, depending on the context. Even for an irregular sequence, which has no definitive rhythm, or just a single event, there could be both temporal and frontal activity by means of direct vestibular activation if the stimulus is above threshold. However, the question of whether rhythm perception is possible without any motor or pre-motoric activity is one which will require further experimental investigation.

## Conflict of Interest Statement

The authors declare that the research was conducted in the absence of any commercial or financial relationships that could be construed as a potential conflict of interest.
